# Synthesis, Photo-Characterizations, and Pre-Clinical Studies on Advanced Cellular and Animal Models of Zinc(II) and Platinum(II) Sulfonyl-Substituted Phthalocyanines for Enhanced Vascular-Targeted Photodynamic Therapy

**DOI:** 10.1021/acsami.4c04138

**Published:** 2024-09-06

**Authors:** Paweł Repetowski, Marta Warszyńska, Anna Kostecka, Barbara Pucelik, Agata Barzowska, Atefeh Emami, Ümit İşci, Fabienne Dumoulin, Janusz M. Dąbrowski

**Affiliations:** † Faculty of Chemistry, Jagiellonian University, Kraków 30-387, Poland; ‡ Doctoral School of Exact and Natural Sciences, Jagiellonian University, Kraków 30-348, Poland; § Małopolska Centre of Biotechnology, Jagiellonian University, Kraków 30-387, Poland; ∥ Łukasiewicz Research Network—Kraków Institute of Technology, Kraków 30-418, Poland; ⊥ Faculty of Technology, Department of Metallurgical & Materials Engineering, Marmara University, Istanbul 34722, Türkiye; # Faculty of Engineering and Natural Sciences, Department of Biomedical Engineering, Acıbadem Mehmet Ali Aydınlar University, Ataşehir, Istanbul 34752, Türkiye

**Keywords:** advanced cellular models, anticancer activity, organoids, photodynamic therapy (PDT), phthalocyanines, reactive oxygen species (ROS), vascular-targeted photodynamic therapy (V-PDT)

## Abstract

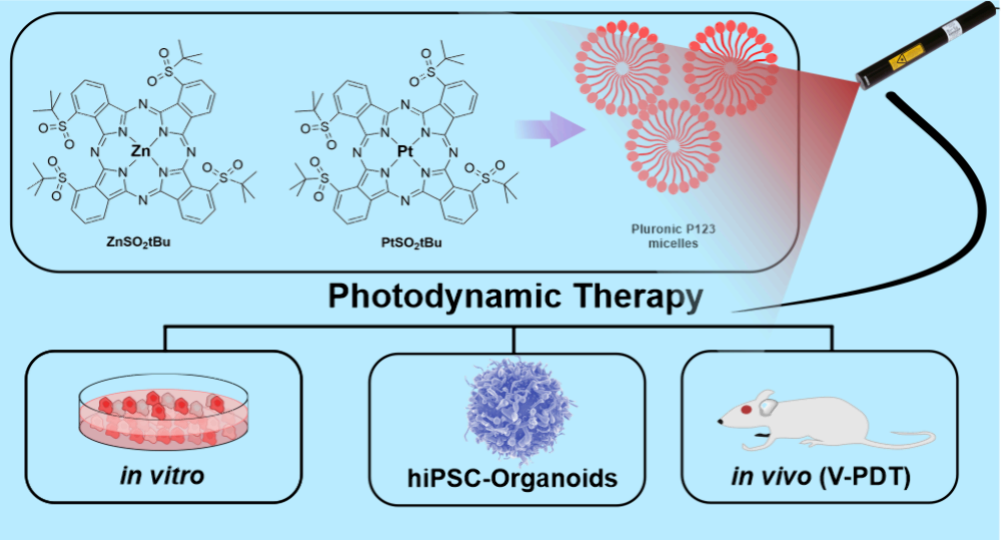

Two phthalocyanine derivatives tetra-peripherally substituted with *tert*-butylsulfonyl groups and coordinating either zinc(II) or platinum(II) ions have been synthesized and subsequently investigated in terms of their optical and photochemical properties, as well as biological activity in cellular, tissue-engineered, and animal models. Our research has revealed that both synthesized phthalocyanines are effective generators of reactive oxygen species (ROS). **PtSO**_**2**_***t*Bu** demonstrated an outstanding ability to generate singlet oxygen (Φ_Δ_ = 0.87–0.99), while **ZnSO**_**2**_***t*Bu** in addition to ^1^O_2_ (Φ_Δ_ = 0.45–0.48) generated efficiently other ROS, in particular ·OH. Considering future biomedical applications, the affinity of the tested phthalocyanines for biological membranes (partition coefficient; log *P*_ow_) and their primary interaction with serum albumin were also determined. To facilitate their biological administration, a water-dispersible formulation of these phthalocyanines was developed using Pluronic triblock copolymers to prevent self-aggregation and improve their delivery to cancer cells and tissues. The results showed a significant increase in cellular uptake and phototoxicity when phthalocyanines were incorporated into the customizable polymeric micelles. Moreover, the improved distribution in the body and photodynamic efficacy of the encapsulated phthalocyanines were investigated in hiPSC-delivered organoids and BALB/c mice bearing CT26 tumors. Both photosensitizers exhibit strong antitumor activity. Notably, vascular-targeted photodynamic therapy (V-PDT) led to complete tumor eradication in 84% of **ZnSO**_**2**_***t*Bu** and 100% of **PtSO**_**2**_***t*Bu-**treated mice, and no recurrence has so far been observed for up to five months after treatment. In the case of PtSO_2_*t*Bu, the effect was significantly stronger, offering a wider range of light doses suitable for achieving effective PDT.

## Introduction

Photodynamic therapy (PDT) is a photochemistry-based modality applied to treat solid tumors but also is used in dermatology (nonmelanoma skin cancers, acne vulgaris), ophthalmology (age-related macular degeneration, AMD), and in the treatment of localized infections,^1−3^ especially those caused by multidrug-resistant microorganisms.^4,5^ At the core of PDT are three essential elements—light, molecular oxygen, and a photosensitizer (PS). Separately, at the doses used, they are nontoxic to the organisms, but their proper combination leads to the generation of reactive oxygen species (ROS) which, due to their highly cytotoxic properties, are effective weapons inflicting oxidative damages.^6^ PDT has been successfully clinically validated as a minimally invasive therapeutic strategy that results in the induction of many biochemical mechanisms leading to damage to tumor blood vessels,^7,8^ death of transformed tumor cells, induction of local inflammation, and ultimately long-term systemic immune response.^9,10^ These events can be the consequence of two major photochemical mechanisms (Type I and Type II mechanisms). Noteworthy, the Type I mechanism occurs even under hypoxic conditions. The PS reacts with an organic molecule within the tumor tissue to form the corresponding radicals. The produced anion radicals, in most cases, react immediately with the oxygen molecule, resulting in the formation of a mixture of oxygen intermediates. The resulting superoxide ion (O_2_·^–^) initiates a cascade of reactions leading to the formation of more or less ROS, including hydrogen peroxide (H_2_O_2_) and then hydroxyl radical (·OH).^7,11^ Type II mechanism takes place under conditions of reasonable oxygen availability in illuminated tissues. It involves the direct transfer of energy from the PS in its triplet excited state to molecular oxygen in the ground state, resulting in the quenching of the excited triplet state of the PS with the simultaneous generation of singlet oxygen (^1^O_2_).^12^ The photodynamic effect is largely mediated by the lower-energy form of singlet oxygen (^1^Δ_g_)^7^ due to its metastability and relatively long half-life. The photogenerated ROS are characterized by a pronounced reactivity toward key biomolecules (unsaturated lipids, nucleic acids, and amino acid residues in proteins), but due to their reduced lifetime in the biological media, the photodynamic effect is limited to sites of their production.^13^ A situation in which both electron/hydrogen atom transfer (Type I) and energy transfer (Type II) mechanisms are involved in the photodynamic effect is considered the most desirable.^7^

In addition to the efficient ROS generation, an optimal PS should exhibit several key properties.^7^ Its synthesis should be carried out using available precursors and ensure the reproducibility of the synthesis. It should absorb light in the range of phototherapeutic window (630–850 nm), where endogenous dyes and water do not absorb. Moreover, it is well established that shorter wavelengths are the most scattered by tissues and their penetration is limited, while on the other hand, low-energy photons (λ > 850 nm) are less effective in generating ROS due to thermal effects caused by fast nonradiative transitions and a narrow energy gap. Most importantly, good PSs should exhibit a sufficiently long triplet state lifetime to generate these species with high quantum yields.^14,15^

Depending on the desired effect, the time between drug administration, and the duration of the drug-to-light interval (DLI), PDT can be divided into vascular-targeted photodynamic therapy (V-PDT) and cellular-targeted photodynamic therapy (C-PDT). V-PDT stands out as a potent therapeutic strategy utilizing various PSs to effectively combat well-vascularized tumors.^16,17^ This approach is designed to disrupt tumor vessels, thereby restricting the tumor’s nutrient supply and managing its growth, ideally leading to its complete eradication. V-PDT has been demonstrated to induce an anticipated decrease in tumor oxygenation or an increase in hypoxia.^17,18^ The key feature of V-PDT lies in the prompt application of light immediately after PS administration (DLI = 10–15 min), while it remains within the vascular compartment of the tumor tissue. Hydrophilic compounds such as Tookad@Soluble and TPPS are used as PSs in V-PDT, which bind mainly to albumin after intravenous (i.v.) administration and often show limited photostability.^10^

In contrast, C-PDT specifically aims at selectively obliterating tumor tissue while preserving normal tissue integrity. However, an inherent challenge of PDT is the potential occlusion of blood vessels, inducing hypoxia shortly after initiation. The reversibility of this effect hinges on the PS concentration in the tissue and the applied light dose. Even if vessels initially appear intact and seem to function normally post-treatment, irreversible vascular damage may manifest within hours, resulting in hemorrhage and tissue necrosis. When PDT specifically targets tumor cells, the constrained oxygen consumption may paradoxically lead to enhanced oxygenation, attributed to reduced metabolic oxygen consumption by the cells.^19^ C-PDT extends the DLI to 24, 72, or even 96 h to exploit the PS accumulation in tumor cells and its faster clearance from normal tissues. As a PS in C-PDT are used hydrophobic compounds, such as Temoporfin and unsubstituted **ZnPc**, which require a stabilizing carrier like liposomes or polymeric micelles. This nuanced understanding of the V-PDT and C-PDT mechanisms underscores their potential in tailoring therapeutic interventions for effective tumor management.

Phthalocyanines are a promising group of PSs, as they absorb light in the near-infrared part of the spectrum.^20,21^ Unfortunately, phthalocyanines are prone to aggregation and have usually weak solubility in aqueous solutions.^22,23^ To prevent these phenomena, phthalocyanines containing various substituents are being synthesized, which guarantee better solubility in aqueous media and reduced aggregation in the blood when administered intravenously.^24^ Another option to increase the biocompatibility of phthalocyanines is their encapsulation into suitable biologically compatible carriers to ensure an efficient and selective drug delivery system into the body.^24,25^ Literature reports about the use of nanoparticles,^20,21,26−28^ emulsifiers (including Cremophor EL or Solutol),^22,23^ as well as other lipid-based carriers such as liposomes, or the incorporation of the drug into low-density lipoprotein (LDL).^24,29^ Currently, great therapeutic utility is attributed to copolymeric micelles,^24,25,30^ among which Pluronic has been widely acknowledged.^31^ It is a triblock copolymer made of two hydrophilic polyethylene glycol (PEG), poly(ethylene oxide) (PEO) fragments, and one hydrophobic polypropylene glycol (PPG) fragment. They can self-organize in an aqueous environment and form micelles encapsulating the compound, which reduces the aggregation of molecules, as well as increases their solubility in polar solvents, and are known for their biocompatibility and innocuousness compared to widely used Cremophor.

Besides the modulation of the water-solubility and aggregation, the photochemical properties of phthalocyanines can be affected by their substitution^32−35^ and metalation pattern.^36^ The methylsulfonyl substitution pattern has produced highly efficient photosensitizing phthalocyanines.^37^ Heavy atoms such as iodine^38^ or heavy metal ions are known to increase the intersystem crossing that favors ROS generation.^39^ However, the insertion of platinum or palladium at the center of a phthalocyanine can have undesired effects such as a blue shift of their maximum absorption and increased aggregation.^36^ To investigate the effect of Pt(II) metalation compared to classical Zn(II) metalation and to benefit from the sulfonyl substitution pattern while limiting the aggregation, two phthalocyanines, **ZnSO**_**2**_***t*Bu** and **PtSO**_**2**_***t*Bu**, have been designed. The substituents are inserted in α/nonperipheral positions of the phthalocyanine ring, and the *tert*-butyl sulfonyl moieties have been selected for their bulkiness, aiming at increasing the solubility. For comparison, studies were also performed on commercially available unsubstituted **ZnPc**. A detailed photocharacterization study has been completed together with the determination of the *n*-octanol/water partition coefficient (log *P*_OW_) and the evaluation of their interactions with plasma proteins. Next, in vitro studies on various cell lines and human-induced pluripotent stem cells (hiPSC)-derived colonic organoids have been performed to assess the accumulation of the tested compounds in cancer cells and organoids as well as cyto- and photocytotoxicity. Moreover, we conducted in vivo experiments on BALB/c mice bearing colon tumors (CT26), comparing **ZnSO**_**2**_***t*Bu** and **PtSO**_**2**_***t*Bu** with commercially available **ZnPc** in two approaches: V-PDT and C-PDT, after previous noninvasive fluorescence imaging.

## Results and Discussion

### Synthesis

**Scheme 1:**
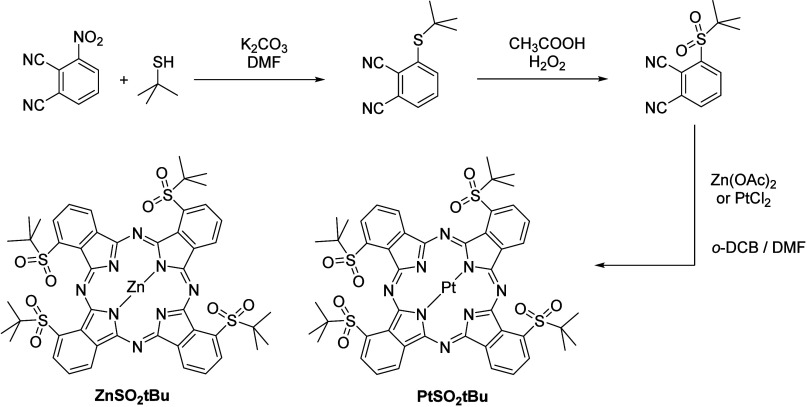
Scheme of the Synthesis of Tetra Nonperipheral *tert*-Butylsulfonyl-Substituted Phthalocyanines **ZnSO**_**2**_***t*Bu** and **PtSO**_**2**_***t*Bu**

The synthesis of **ZnSO**_**2**_***t*Bu** and **PtSO**_**2**_***t*Bu** is straightforward and includes three high-yielding steps (Scheme 1). First, *tert*-butanethiol reacts with 3-nitrophthalonitrile at room temperature in DMF in the presence of K_2_CO_3_ in a 75% yield. The resulting thioether functional group is oxidized in refluxing acetic acid by hydrogen peroxide, still with a very high yield (92%). An important parameter must then be taken into account during the last reaction, which is the cyclotetramerization of this late phthalonitrile. As the sulfonyl functional group is sensitive to nucleophilic substitutions, the classical solvents used for this reaction cannot be employed, as they are usually high-boiling point alcohols in basic conditions (dimethylaminoethanol or pentanol/DBU) which attack the sulfonyl functional group.^40^ This is why sulfonylphthalonitriles are generally cyclotetramerized in a mixture of DMF and *ortho*-dichlorobenzene in the presence of the desired metal salt. The yields of the synthesis of both phthalocyanines are similar and very high (>30%), which is an excellent result.

### Photophysics and Photochemistry

#### Ground-State Electronic Absorption Spectra

**Figure 1:**
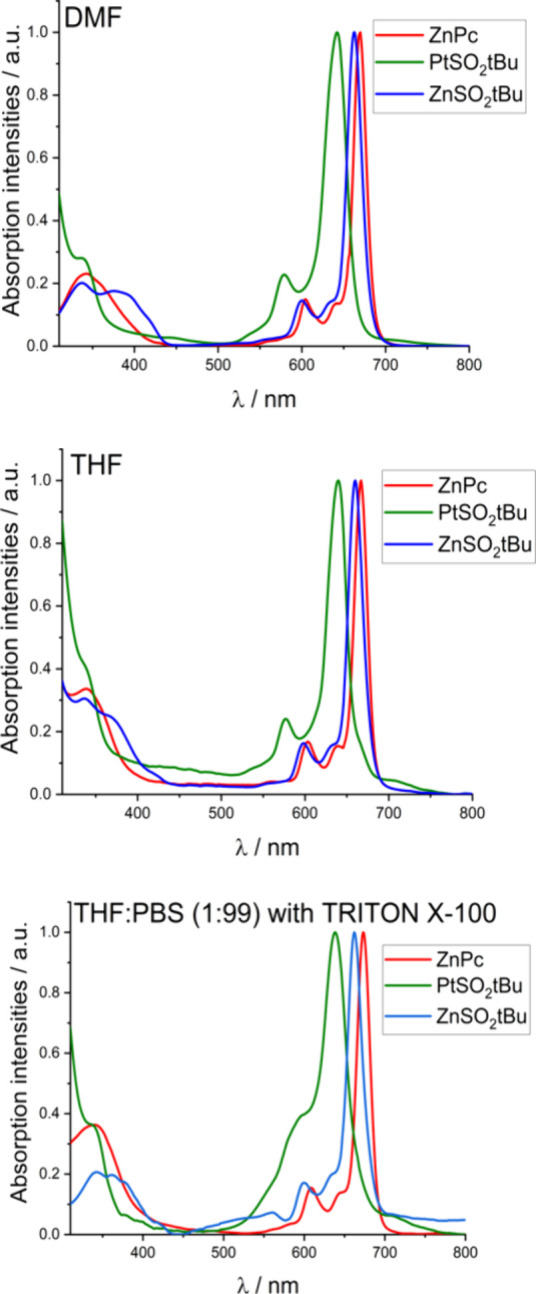
Electronic absorption spectra of **ZnPc**, **ZnSO**_**2**_***t*Bu**, and **PtSO**_**2**_***t*Bu** measured in THF, DMF, and THF:PBS (1:99) with 0.1% TRITON X-100 at room temperature.

The optical properties of **ZnSO**_**2**_***t*Bu** and **PtSO**_**2**_***t*Bu** have been studied in various solvents, and unsubstituted **ZnPc** was used as a standard. Among the possible solvents, tetrahydrofuran (THF) and *N*,*N*-dimethylformamide (DMF) proved to be the most appropriate due to their good solubility, nonaggregation, and relevant electronic absorption. The obtained data are shown in Figure 1, and the relevant properties are summarized in Table 1.

**Table 1:** Optical, Photophysical, and Photochemical Properties of Phthalocyanines **ZnSO**_**2**_***t*Bu**, **PtSO**_**2**_***t*Bu**, and **ZnPc** in DMF

compound	λ_max_/nm	ε × 10^3^/M^−1^·cm^–^^1^	log ε × 10^3^/log (M^−1^·cm^–1^)	λ_em_/nm	Δλ_em_^St^/ nm	τ_F_/ns	Φ_F_	τ_T_/ns	*k*_q_/M^–1^ s^–1^
**ZnSO_2_*t*Bu**	660	162.9	5.09	667	7	2.82 ± 0.02	0.172 ± 0.014	348	2.05 × 10^9^
**PtSO_2_*t*Bu**	640	82.0	4.41	665	25	2.75 ± 0.05	0.00012 ± 0.00002	275	2.15 × 10^9^
**ZnPc**	666	207.7	5.34	673	7	3.61 ± 0.05	0.229 ± 0.012	335	2.13 × 10^9^

Two characteristic absorption bands can be distinguished in the electronic absorption spectra of the studied phthalocyanines: the Q-band at about 600–700 nm, and the Soret band at ca. 350 nm, which is less intense and wider than the Q-band. Both bands are the result of π → π*-electron transitions occurring in the phthalocyanine molecule. The molar absorption coefficients (ε) were determined for both PSs by plotting the absorption intensities in the Soret and *Q* bands as a function of the concentration. The linearity of Lambert–Beer’s law is preserved for the entire concentration range. The calculated values of ε and log ε are summarized in Table 1.

The presence of the sulfonyl substituents in a nonperipheral position does not significantly affect the maximum of the Q-band for **ZnSO**_**2**_***t*Bu** compared to **ZnPc**, with a slight blueshift only. The effect of the metal ions is more important, with nearly 20 nm of difference between the absorption for **ZnSO**_**2**_***t*Bu** (660 nm) compared to **PtSO**_**2**_***t*Bu** (641 nm), a previously observed phenomenon.^36^ Aggregation is the result of coplanar interactions between nearly flat macrocyclic rings. It involves molecules joining together first to form dimers and then into higher-order complexes. The degree of aggregation depends on external factors (temperature, type of solvent, and phthalocyanine concentration) and structural features (type of substituents and coordinated metal ions). Unsubstituted phthalocyanines have a strong tendency to aggregate in most of the solvents. As a result, there is a reduction in the intensity and broadening of the Q-band. This effect is seen on the spectrum of **PtSO**_**2**_***t*Bu** in the PBS:THF with the addition of TRITON X-100. (Figure 1). The addition of 1% THF and 0,1% TRITON X-100 is a gold standard for reducing the aggregation of phthalocyanines in PBS, but on the other hand, this system cannot be used for in vitro studies. While this concentration of THF is not toxic to cells, however, the usage of TRITON X-100 in in vitro studies should be avoided, as it increases the permeabilization of the cell membrane in low concentrations or may lead to cell lysis in higher concentrations.^41^

#### Photophysical Properties

Fluorescence steady-state measurements provide information about the fluorescence excitation and emission wavelengths, the Stokes shifts, and the fluorescence quantum yields. These properties are summarized in Table 1. Steady-state fluorescence of phthalocyanines was recorded in THF, DMSO, and ethanol and shown in Figure S1 along with absorption spectra in the same solvent. A good PDT PS should have a low fluorescence quantum yield but should not be completely lacking in fluorescence properties, due to the possibility of its easy detection and monitoring the progress of therapy. However, a more important photochemical property is the occupation of long-lived triplet states, a competing phenomenon that is crucial for the subsequent generation of ROS. Although electronic transitions from the singlet to triplet excited states are forbidden, excitation of molecules to triplet states is possible due to the heavy atom effect, which leads to the so-called spin–orbit coupling. The heavy atom effect on the fluorescence quantum yield is particularly evident in the case of **PtSO**_**2**_***t*Bu** phthalocyanine, in which Φ_F_ value is negligible. Small values of the average fluorescence lifetime (τ_F_) are associated with a low quantum yield (Φ_F_). **ZnPc** has the longest fluorescence lifetime, while the values for both substituted compounds (**ZnSO**_**2**_***t*Bu** and **PtSO**_**2**_***t*Bu**) are correspondingly lower, showing that this parameter can also be influenced by the presence of sulfonyl substituents. The time-resolved fluorescence fading profile and response function of the IRF (instrument response function) apparatus are presented (Figure S2a), and an appropriately fitted exponential model and the distribution of the normalized weighted difference function (residuals) for the tested compounds of the phthalocyanine group (Figure S2b) are shown to assess the quality of the fit.

#### Triplet State Lifetimes

**Figure 2:**
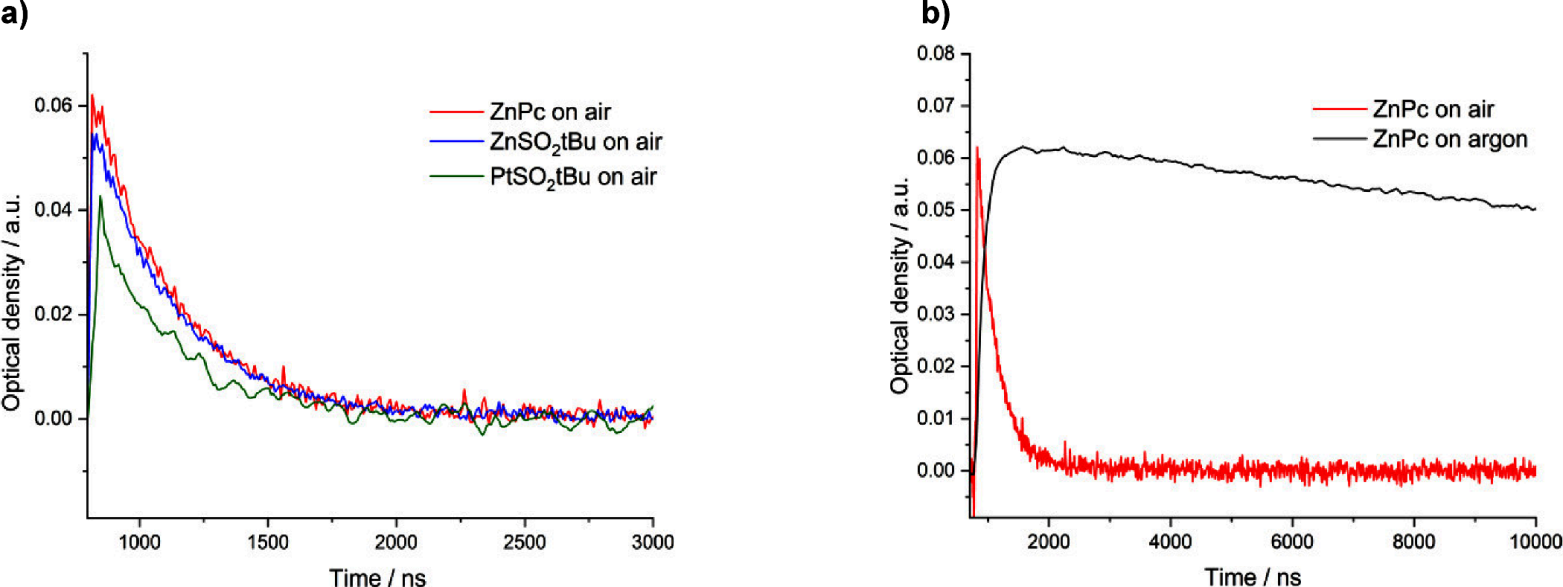
(a) Triplet state decays of the **ZnPc**, **ZnSO**_**2**_***t*Bu**, and **PtSO**_**2**_***t*Bu** in the air-saturated DMF solutions measured at 490 nm by laser flash photolysis at 20 °C with λ_ex_ = 355 nm. (b) Influence of the presence of molecular oxygen on the decay of **ZnPc** triplet state (solutions were purged with argon).

Based on previous studies of the transient absorption of **ZnPc**,^42^ laser flash photolysis measurements were performed for **ZnPc**, **ZnSO**_**2**_***t*Bu**, and **PtSO**_**2**_***t*Bu** under normal atmospheric conditions. Transient decay times in the presence of oxygen are independent of the monitored wavelength and are typical of triplet states transferring energy quantitatively to molecular oxygen.^43^Table 1 presents the determined triplet state lifetimes for all three PSs. When the phthalocyanine solution remains in equilibrium with oxygen in the air, the decay remains monoexponential with lifetimes extending to the nanosecond range. Figure 2a illustrates the decay of the triplet state signal for **ZnPc**, **ZnSO**_**2**_***t*Bu**, and **PtSO**_**2**_***t*Bu** in DMF. The triplet state lifetimes for studied phthalocyanines closely resemble those reported for halogenated porphyrins and are even longer than those for halogenated bacteriochlorins already applied in various models in vivo^44−46^ and in clinical trials.^47^ The shorter triplet state lifetime observed for **PtSO**_**2**_***t*Bu** is consistent with literature data, as it is well-established that the larger the orbital spin coupling constant, the shorter the triplet state lifetime (so-called heavy atom effect).^14^ However, this is still a sufficient time for the effective generation of singlet oxygen. Furthermore, following argon saturation, the triplet state lifetimes for each compound were notably prolonged. Specifically, the lifetime for **ZnPc** increased from 335 ns to 33.11 μs, for **PtSO**_**2**_***t*Bu** from 275 ns to 1.55 μs, and most notably for **ZnSO**_**2**_***t*Bu** from 348 ns to 50.28 μs. It is crucial, as the long-lived triplet states provide high sensitivity to the PSs concerning the presence of quenching species in the environment. For **ZnPc**, the decay of the triplet state signal in an argon-saturated solution is shown in Figure 2b (and for **ZnSO**_**2**_***t*Bu** and **PtSO**_**2**_***t*Bu**, it is shown in Figure S4), clearly confirming that the presence of oxygen reduces significantly the triplet lifetime. The rate constant (*k*_q_) for energy transfer from the PS’s triplet state to molecular oxygen can be determined from triplet lifetimes in the absence (τ_T_^0^) and presence of oxygen (τ_T_), using the provided relationship (1):
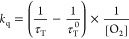
1

#### Photostability

**Figure 3:**
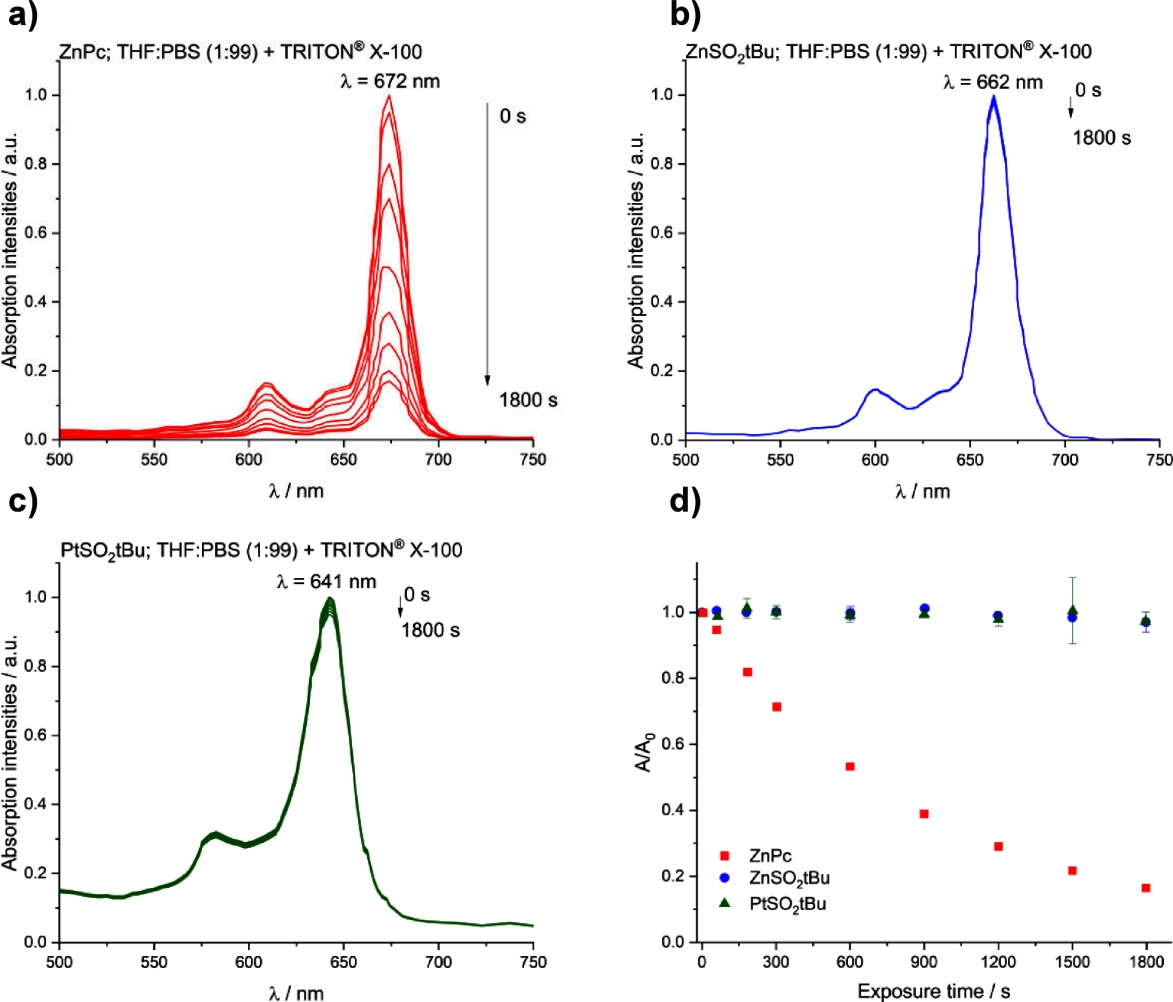
Changes in absorption intensity for (a) **ZnPc**, (b) **ZnSO**_**2**_***t*Bu,** (c) **PtSO**_**2**_***t*Bu** in THF:PBS (1:99), exposure time 30 min; (d) correlation *A*/*A*_0_ = *f*(*t*) during photostability testing of **ZnPc** compounds in THF:PBS (1:99) with 0.1% TRITON X-100, laser diode 635 ± 20 nm, light intensity 17 mW, exposure time 30 min, cutoff filter λ < 550.

Phthalocyanines under visible light and in the presence of molecular oxygen can undergo photobleaching. An evaluation of the photostability of the tested phthalocyanines was carried out in various solvents. Of the three compounds tested, only **ZnPc** showed a significant decrease in the color intensity of the solution, confirmed by the disappearance of the Q-band as the sample was exposed to light. For substituted derivatives **ZnSO**_**2**_***t*Bu** and **PtSO**_**2**_***t*Bu**, no change in the Q-band intensity was observed. In addition, the shape of the spectrum did not change after irradiation, only the intensity of the band’s absorbance (Figure 3). **ZnSO**_**2**_***t*Bu** and **PtSO**_**2**_***t*Bu** are characterized by significantly improved photostability compared to **ZnPc**, an effect that can be attributed rather to the presence of the sulfonyl substituents than to the coordinated metal, in line with previous observations.^32^ This is an excellent finding, as only a sufficiently photostable compound can participate in more photocatalytic cycles and thus generate greater amounts of singlet oxygen or other types of ROS.

#### Singlet Oxygen Quantum Yields

**Figure 4:**
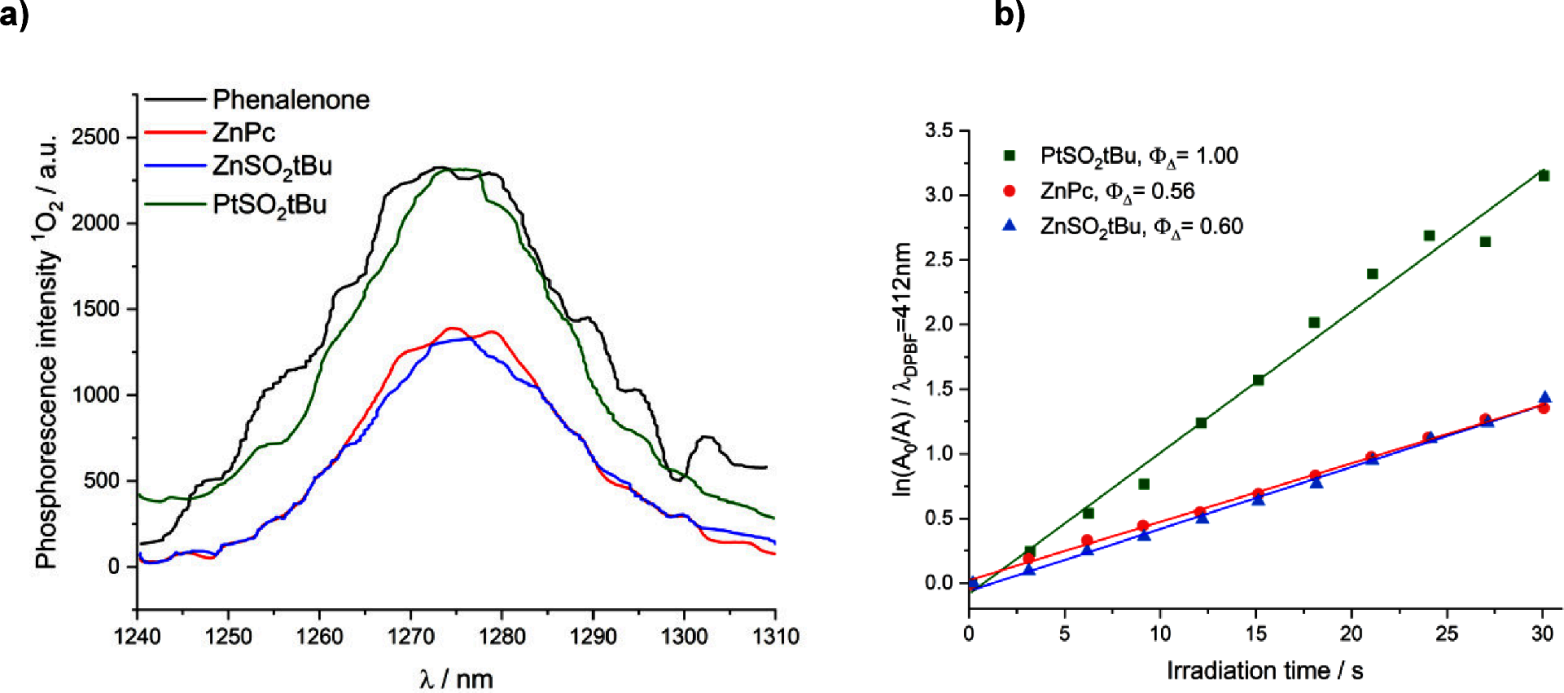
(a) Phosphorescence spectra of singlet oxygen (λ_max_ ∼ 1270 nm) for phenalenone and the tested compounds of the phthalocyanine group in DMF (λ_ex_ = 350 nm) plotted for the single-point method at voltages on the detector equal to 1600 V. (b) Dependence of ln(*A*_0_/*A*) on exposure time for a mixture of DPBF with **ZnSO**_**2**_***t*Bu**, **PtSO**_**2**_***t*Bu**, and **ZnPc**.

Different methods can be used to detect singlet oxygen and monitor its generation. The so-called indirect method is based on the use of a chemical quencher of singlet oxygen, 1,3-diphenylisobenzofuran (DPBF), in organic solvents. A decrease in the intensity of the absorption band of DPBF at λ = 414 nm in DMF was observed after red light illumination (635 ± 20 nm). The recorded changes in the shape of the absorption spectra of the mixture of DPBF and phthalocyanine during irradiation are shown in Figure S3. The dependence of ln(*A*_0_/*A*) on the time of radiation exposure, plotted for the tested PSs, is presented in Figure 4b. The values of the quantum yield of singlet oxygen generation determined by the indirect method are summarized in Table 2.

**Table 2:** Singlet Oxygen Quantum Yields (Φ_Δ_) Determined for the Tested Compounds in DMF Using the Indirect Method and the Single-Point Method Based on the Stationary Spectra of Characteristic Singlet Oxygen Phosphorescence

phthalocyanine	Φ_Δ_	
	chemical quenching of DPBF in DMF	phosphorescence of singlet oxygen in DMF
**ZnPc**	0.56 ± 0.06^49^	0.47 ± 0.02
**ZnSO**_**2**_***t*Bu**	0.48 ± 0.01	0.45 ± 0.02
**PtSO**_**2**_***t*Bu**	0.99 ± 0.01	0.87 ± 0.02

Another method involves the detection of characteristic ^1^O_2_ phosphorescence at 1270 nm in DMF (Figure 4 and Table 2). The measurements were also conducted under anaerobic conditions to exclude the potential involvement of phthalocyanine phosphorescence (Figure S5). Under anaerobic conditions, no signal suggesting that the phosphorescence phenomenon of the PS could influence the determined values of the singlet oxygen generation quantum yield was observed. Literature values of Φ_Δ_ determined for **ZnPc** in ethanol using analogous techniques to the present work are also included. The values cited in the literature and those determined for commercially available **ZnPc** are similar, despite the different solvents in which the measurement was performed.

The data obtained from the two different techniques allow ranking of the studied phthalocyanines in terms of singlet oxygen quantum yield values in the following order: **PtSO**_**2**_***t*Bu** > **ZnPc** > **ZnSO**_**2**_***t*Bu**. Comparing the Φ_Δ_ values obtained for the same compounds, but using different methods, one can see their clear overestimation in the case of detection using the chemical quenching technique with DBPF and demonstrating that the more reliable method is the direct detection of ^1^O_2_ luminescence based on the measurement of the characteristic phosphorescence spectrum of singlet oxygen at λ_em_ ∼ 1270 nm. Other researchers have noted comparable fluctuations in the singlet oxygen generation efficiencies when employing distinct approaches. To illustrate, when determining values for **ZnPc** in ethanol, the chemical quenching of the DPBF method yielded a value of 0.53 ± 0.15, whereas the single-point method relying on the stationary spectra of characteristic singlet oxygen emission produced a value of 0.4 ± 0.1.^48^ While the quantum yields of singlet oxygen for both phthalocyanines with Zn(II) metalation are very similar, **PtSO**_**2**_***t*Bu** exhibits a much higher capacity to generate ^1^O_2_, in line with the expected heavy atom effect and its significant effect on increasing the occurrence of intersystem crossing, increasing the generation of singlet oxygen.

#### Detection of Type I Photoproducts by Fluorescent Probe

**Figure 5:**
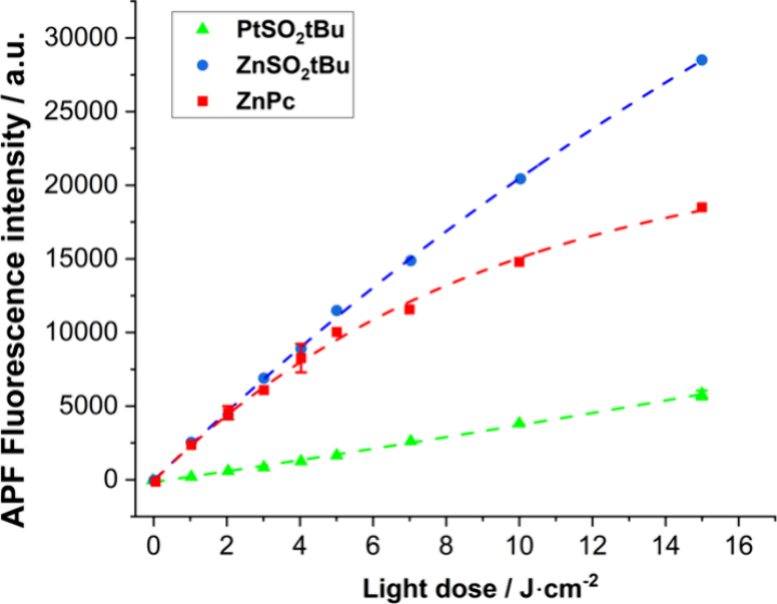
Generation of oxygen radicals by **ZnSO**_**2**_***t*Bu**, **PtSO**_**2**_***t*Bu**, and **ZnPc**. Fluorescence generated from APF probe (15 μM) during irradiation of each photosensitizer solution (10 μM), THF:PBS ca. 1:99 v/v.

ROS can function as components of signal transduction or react with biomolecules to cause oxidative damage to cells. Detection of other than singlet oxygen ROS (products of Type I photochemical reaction) was carried out using 3′-(*p*-aminophenyl)fluorescein (APF), relatively specific to hydroxyl radicals.^50^ Changes in the probe fluorescence intensity during irradiation are presented in Figure 5. Detection of ·OH was carried out using PSs of the same concentration (3 μM) and identical absorbance intensity in the Q-band (*A* ∼ 0.25). There was an increase in fluorescence intensity with light exposure time for each phthalocyanine. The fluorescence intensity of APF compared to **ZnPc** is higher for the phthalocyanine **ZnSO**_**2**_***t*Bu** and lower for **PtSO**_**2**_***t*Bu**. In addition, changes in APF fluorescence intensity upon exposure to red light (635 ± 20 nm) show a rather linear trend (Figure 5). These results indicate that although **ZnSO**_**2**_***t*Bu** does not exhibit as high singlet oxygen quantum yield as **PtSO**_**2**_***t*Bu**, it can further be an effective PS in biological research, as it generates ·OH more efficiently than the platinum derivative. This property is particularly important in the treatment of hypoxic tumors, where access to molecular oxygen is limited. **ZnSO**_**2**_***t*Bu** is likely to act through both photochemical mechanisms involving electron/hydrogen atom transfer and direct energy transfer, while **PtSO**_**2**_***t*Bu** is an extremely strong, efficient type II PS.

#### *n*-Octanol/PBS Partition Coefficients

The values of the partition coefficients for the tested compounds were determined using the modified shake-flask method (Figure S6). The obtained log *P*_OW_ values determined for **ZnPc** and **ZnSO**_**2**_***t*Bu** (Table 3) meet Lipinski’s rules.^51^ The presence of the sulfonyl moieties introduced on the phthalocyanine ring lowered the log *P*_OW_ values of the compounds. In the case of **PtSO**_**2**_***t*Bu**, it was not possible to accurately determine the log *P*_OW_ due to its negligible fluorescence. Nevertheless, we assume that the metal ion does not significantly influence the log *P*_OW_ value, and it can be established that the log *P*_OW_ of **PtSO**_**2**_***t*Bu** is similar to that of **ZnSO**_**2**_***t*Bu**. The estimation of the log *P*_OW_ value allows a rational choice of the appropriate formulation for further biological studies.

**Table 3:** Partition Coefficients, Binding Ability, and Fluorescence Quenching Data for BSA Interaction, Binding Constants (*K*_b_) Calculated for Studied Phthalocyanines

phthalocyanine	log *P*_OW_	*K*_sv_ × 10^5^ [M^–1^]	*k*_qBSA_ × 10^13^ [M^–1^ s^–1^]	log *K*_b_	*K*_b_ × 10^4^ [M^–1^]	*n*
**ZnPc**	4.7	3.12 ± 0.23	3.12	4.05 ± 0.38	1.12	∼1
**ZnSO**_**2**_***t*Bu**	2.22	3.20 ± 0.15	3.20	5.42 ± 0.67	26.30	∼1
**PtSO**_**2**_***t*Bu**		5.27 ± 0.26	5.27	4.46 ± 0.88	2.88	∼1

#### Interaction with Bovine Serum Albumin (BSA) Plasma Proteins

When the PS enters the bloodstream, it binds to plasma proteins and is then released into tissues. Fluorescence quenching and Scatchard analysis were used to establish the interaction of phthalocyanines with BSA. Tryptophan residues present in albumin, which fluoresce at a wavelength of about 350 nm, were used to test the fluorescence of the albumin. Upon binding, BSA and phthalocyanine undergo mutual fluorescence quenching, allowing for quantification of binding. Emission spectra recorded for a fixed concentration of BSA during a series of titrations with the tested PSs from the phthalocyanine group are presented in Figure S7. The apparent shift of the emission maximum toward shorter wavelengths from 350 to 335 nm is most likely due to a change in the conformation of the protein due to modification of the microenvironment around the protein after the introduction of the corresponding compounds.

**Figure 6:**
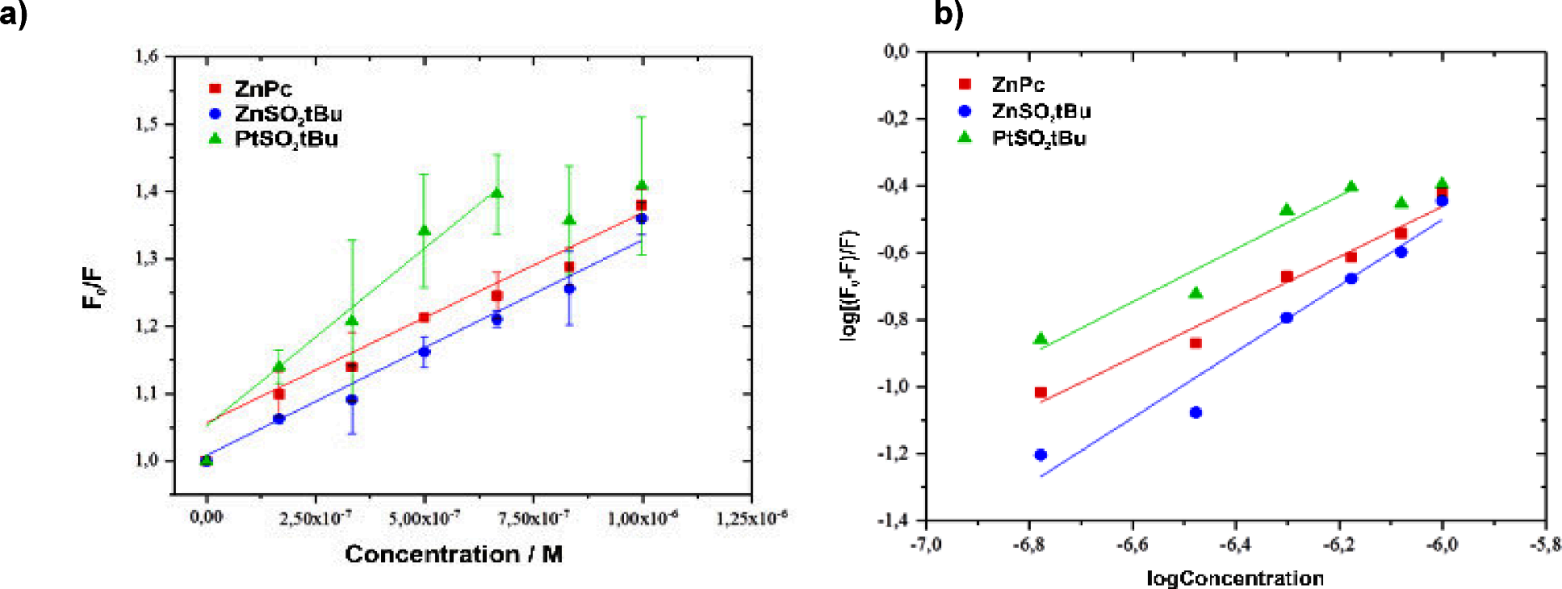
(a) Stern–Volmer plots of **ZnPc**, **ZnSO**_**2**_***t*Bu**, and **PtSO**_**2**_***t*Bu** quenching of BSA. (b) Scatchard analysis plots.

Figure 6 shows a Stern–Volmer plot presenting the dependence of the fluorescence intensity ratio as a function of the PS’s concentration. The values of *K*_SV_ and *k*_q_ determined for each compound are summarized in Table 3. Among the analyzed PSs, the highest value of the Stern–Volmer constant is characterized by **PtSO**_**2**_***t*Bu**. Determined from the ratio *K*_SV_/τ_0_, the values of the bimolecular quenching rate constant allowed the characterization of the mechanism of fluorescence quenching in the studied BSA-Pc systems. In each of the analyzed cases, the obtained values of bimolecular quenching rate constants were three orders higher than the maximum value of *k*_q_ allowed for diffusion-controlled dynamic quenching (10^10^ M^–1^ s^–1^). Therefore, the more likely mechanism is static quenching, which involves the formation of a nonfluorescent complex in the ground state of the fluorophore.^52^ In addition, using Scatchard analysis, the values of the binding constant (*K*_b_) and the number of binding sites on BSA (*n*) were determined (Figure 6). The obtained values of *n* are close to 1, which is probably due to the formation of Pc-protein adducts in a ratio of 1:1. Thus, the most likely site of single bond formation will be the hydrophobic pocket with Trp-214 in the IIA subdomain of the protein.^53^ The determined values of binding constants (*K*_b_) for the substituted phthalocyanines appear to be close to typical *K*_b_ values reported by the literature and characteristic of compounds from the phthalocyanine group (10^4^–10^6^ M^–1^).^54^ However, some disagreement appears in the case of commercial **ZnPc**, for which the quoted *K*_b_ values are rather one or two orders higher. Comparatively, the highest value of the binding constant was determined for **ZnSO**_**2**_***t*Bu**, while successively lower values were determined for **PtSO**_**2**_***t*Bu**. The obtained *K*_b_ values may differ slightly from the estimated values, as aggregates are formed in aqueous solutions, which may underestimate the value of the binding constant. Therefore, when analyzing the Pc-protein interaction, the dissociation constant of the formed aggregates should also be taken into consideration.^55^ The most lipophilic **ZnPc** is characterized by the lowest *K*_b_ value, and **ZnSO**_**2**_***t*Bu** has a high binding constant value and a rather low value of log *P*_OW_ ∼ 2. In the case of **PtSO**_**2**_***t*Bu**, it is not possible to consider pharmacological parameters analogously due to the unreliability of the determined value of log *P*_OW_. The results were described in the previous section (interaction with BSA plasma protein).

#### Micellar Formulation of Phthalocyanines

Due to the limited solubility of the studied compounds and their tendency to aggregate in aqueous solution, they were encapsulated in Pluronic-based micelles. Building on the findings of a previous publication,^56^ several Pluronic-based copolymers, including P123, F127, and a mixture of P123/F127 (2:1), were investigated to identify the most suitable carrier for each phthalocyanine. Pluronic P123, characterized by intermediate PPO block length and moderately hydrophobic structure, appears to be an ideal copolymer for facilitating the effective delivery of **ZnSO**_**2**_***t*Bu** and **PtSO**_**2**_***t*Bu**, as confirmed by test results. Additionally, encapsulating phthalocyanine in micelles ensures protection against unfavorable interactions in the biological environment, ensuring the stability and efficacy of the drug.^57^ Particle diameter was assessed by utilizing dynamic light scattering (DLS) to study the ability of Pluronic to inhibit aggregation in PBS at PDT-relevant concentrations. As depicted in Figure S8, the nanoparticle size in the formulations of **ZnSO**_**2**_***t*Bu-P123** and **PtSO**_**2**_***t*Bu-P123** closely resembles the size of pure Pluronics nanoparticles in PBS (Table 4). This indicates that the phthalocyanines are effectively dissolved and do not form aggregates in the P123 micelles.

**Table 4:** Size of Phthalocyanine Particles Incorporated in Pluronic® P123 Measured by DLS

	particle diameter/nm
	P123
no PS	20.53
**ZnSO**_**2**_***t*Bu-P123**	27.77
**PtSO**_**2**_***t*Bu-P123**	23.88

## Biological In Vitro Studies

**Figure 7:**
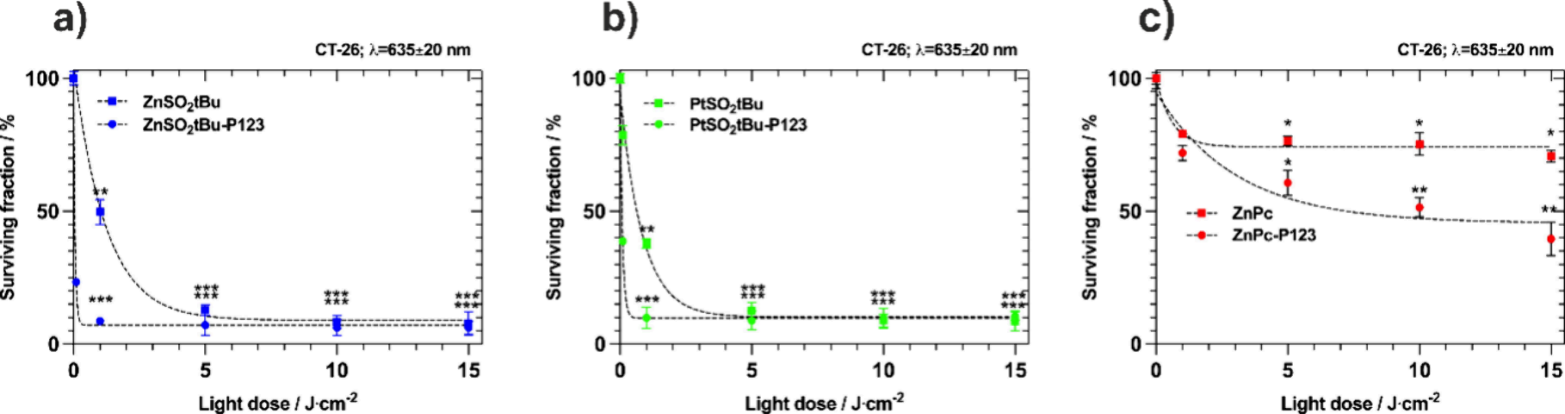
Photodynamic effect of (a) **ZnSO**_**2**_***t*Bu**, **ZnSO**_**2**_***t*Bu-P123**, (b) **PtSO**_**2**_***t*Bu** and **PtSO**_**2**_***t*Bu-P123**, and (c) **ZnPc** and **ZnPc-P123** against CT26 cells. The asterisks denote *p*-values < *0.05, **0.01 compared to control.

The photodynamic effects of **ZnSO**_**2**_***t*Bu**, **ZnSO**_**2**_***t*Bu-P123**, **PtSO**_**2**_***t*Bu** and **PtSO**_**2**_***t*Bu-P123** have been studied against human lung adenocarcinoma (A549), Lewis lung carcinoma (LLC), human breast cancer cells (MCF-7), murine endothelial/vascular epithelium (2H11), Figure S9, and murine colorectal carcinoma (CT26) cell lines (Figure 7). The cell lines used in the study were selected to represent different types of cancer, allowing a comprehensive evaluation of the photodynamic effect on various malignancies. The phototoxicity of PSs in THF:PBS (1:99) and the presence of Pluronic against various cancer cells after irradiation are shown in Figure 7. Research on **PtSO**_**2**_***t*Bu** and **ZnSO**_**2**_***t*Bu** PSs has demonstrated their significant efficacy in photodynamic killing of A549 cells, LLC cells, and CT26 cells compared with other cells studied here. In the case of 2H11 endothelial cells, a minimal photodynamic effect was observed, both for the phthalocyanines themselves and for phthalocyanines encapsulated in Pluronic P123 micelles. Similarly, a diminished photocytotoxicity was also reported in a breast cancer cell line (MCF-7), most probably due to two transmembrane xenobiotic transport proteins, P-glycoprotein (PGP) and multidrug resistance protein (MRP), which are present in MCF-7 cells. They block the transport of xenobiotics, including synthetic phthalocyanine, and prevent these molecules from entering cells.^58^ In this study, unsubstituted **ZnPc** was used as a reference. In our previous investigation, we described the photodynamic effect mediated by **ZnPc** on CT26, A549, and 2H11 cells.^24^ Here we performed the phototoxicity tests to compare **ZnPc** activity with **PtSO**_**2**_***t*Bu** and **ZnSO**_**2**_***t*Bu** against a set of cancer cells and indicated that its encapsulation in Pluronic P123 micelles increases its efficacy. For better comparison, based on phototoxicity data obtained for all studied PSs, we determined the LLD_50_ values (lethal light dose that caused 50% mortality), Table 5.

**Table 5:** LLD_50_ Values Determined for the Investigated Photosensitizers and Formulations

PS	LLD_50_[J/cm^2^]					
cell line	PtSO_2_*t*Bu	PtSO_2_*t*Bu-P123	ZnSO_2_*t*Bu	ZnSO_2_*t*Bu-P123	ZnPc	ZnPc-P123
CT26	1.0	0.1	1.3	0.1	nd (>15)	7.5
A549	1.3	0.5	4.0	1.0	nd (>15)	14
MCF-7	nd (>15)	3.3	nd (>15)	3.2	nd (>15)	13
LLC	1.7	0.8	0.9	0.7	nd (>15)	15
2H11	nd (>15)	nd (>15)	nd (>15)	nd (>15)	nd (>15)	nd (>15)

The results show that the applied micellar formulation increases the efficacy of PDT against all tested cells. The highest lethality was especially achieved with **ZnSO**_**2**_***t*Bu-P123** and **PtSO**_**2**_***t*Bu-P123** against CT26 cancer cells. These results suggest that the efficiency of PDT may depend on the composition of the substance. These discoveries open new perspectives in the PDT field and call for further research to fully understand the mechanisms of action of **ZnSO**_**2**_***t*Bu** and **PtSO**_**2**_***t*Bu**. In vitro studies also confirm the assumptions based on the results of photochemical properties and generation of ROS. Despite the differences in ROS generation (Figures 4b and 5), both sulfonyl-substituted phthalocyanines tested show high photodynamic efficacy against various cancer cells, particularly CT26. CT26 cells serve as a model for aggressive, undifferentiated, and refractory human colorectal cancers (CRCs).^59,60^ Known for its immortality, ease of cultivation, and widespread availability through repositories, CT26 is a frequently utilized model in studies focusing on carcinogenesis and chemotherapeutics.^61−63^ Moreover, its pathobiological characteristics closely resemble those observed in human CRCs.^64^

### **ZnSO_2_*t*Bu** and **PtSO_2_*t*Bu** Cytotoxicity in hiPSC-Derived Colonic Cancer Organoids

**Figure 8:**
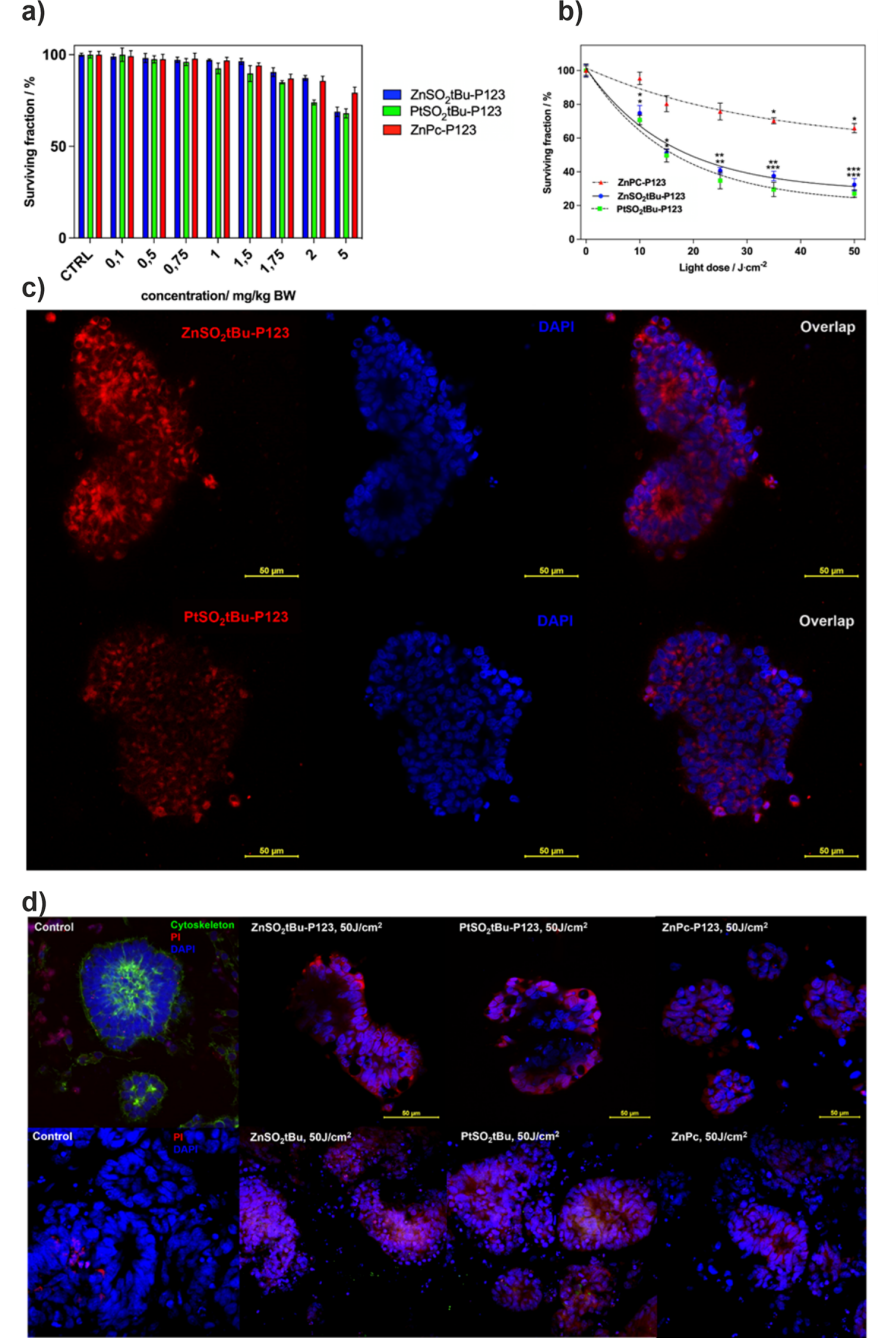
(a) Dark cytotoxicity induced by investigated phthalocyanines on hiPSC-derived CRC organoids. (b) Photodynamic effect induced by studied phthalocyanines against CRC organoids. (c) Fluorescence confocal images of organoids after 24 h incubation with **ZnSO**_**2**_***t*Bu-P123** and **PtSO**_**2**_***t*Bu-P123.** Accumulation of PS is represented in red fluorescence, and nuclei are stained with Hoechst (blue fluorescence). (d) Confocal images of live/death staining with Hoechst/PI for CRC organoids after photodynamic effect (24 h incubation and irradiation with 50 J/cm^2^) mediated by investigated phthalocyanines alone and after their encapsulation in Pluronic P123 micelles.

Organoids are three-dimensional structures that closely resemble organs in structure and functionality. They are derived from stem cells or tissue samples and are grown in the laboratory under controlled conditions. The process of generating organoids usually involves isolating stem cells or specific cell types from the desired organs and providing them with the necessary growth factors and nutrients. These cells self-organize and differentiate to form complex structures that resemble the architecture of the organs they are intended to replicate. They serve as an indirect method of drug screening between in vitro and in vivo research. The development and characterization of the CRC organoid model were previously described by us in the context of nanotoxicity studies.^65^Figure 8a illustrates the cytotoxic impact of **ZnPc-P123**, **ZnSO**_**2**_***t*Bu-P123**, and **PtSO**_**2**_***t*Bu-P123** without the irradiation. All tested phthalocyanines demonstrate minimal dark toxicity in a wide range of concentrations. Only after the highest concentrations of phthalocyanines that correspond to >2 mg/kg BW is the cytotoxicity noticeable (>20%). Fluorescence confocal imaging shows differences in the localization of **ZnSO**_**2**_***t*Bu-P123** and **PtSO**_**2**_***t*Bu-P123** in the organoids (Figure 8c). After a 24 h incubation, Zn-derivative appears to penetrate deep into the organoid structure. The recorded low fluorescence signal in organoids treated with **PtSO**_**2**_***t*Bu-P123** stems from a negligible fluorescence. Consequently, tracking the fluorescence of this phthalocyanine is hindered; therefore, few conclusions about platinum derivative localization can be drawn from those images. After evaluating cytotoxicity in the dark, the photodynamic effect mediated by investigated phthalocyanines was tested. The organoids were incubated with PS’s concentration at 1.5 mg/kg BW for 24 h and then washed with PBS and irradiated with different doses of red light (635 ± 20 nm) (Figure 8b). The obtained data indicate that substituted phthalocyanines show photodynamic activity much higher than that of unsubstituted **ZnPc**. For substituted phthalocyanines, an organoid lethality of 50% was observed at a light dose of 25 J/cm^2^. Higher activity was observed at higher light doses, and for both phthalocyanines, the activity at 50 J/cm^2^ reached about 70%. This value was not obtained for **ZnPc-P123**. For this derivative, the highest organoid mortality of 44% (56% of the surviving fraction) was observed at a light dose of 50 J/cm^2^.

To visualize the phototoxicity, fluorescence confocal microscopy imaging was performed after the photodynamic effect with 50 J/cm^2^. The live/dead staining was prepared with Hoechst 33342 for nuclei staining (living cells, blue fluorescence) and propidium iodide PI (dead cells, red fluorescence). In the control organoid, we also stained the cytoskeleton with phalloidin to determine the organoid structure. In addition, we performed organoid staining to compare the photodynamic effect using encapsulated PS with PS alone (without micellization). The representative images for CRC organoids after the photodynamic effect (50 J/cm^2^, 1.5 mg/kg BW) confirm the higher effectiveness of the **PtSO**_**2**_***t*Bu** and **PtSO**_**2**_***t*Bu-P123** than that for **ZnSO**_**2**_***t*Bu** and **ZnSO**_**2**_***t*Bu-P123**. In the case of Pt-phthalocyanine, the organoid structure after the photodynamic effect appears to be more damaged compared to that of the organoid treated with **ZnSO**_**2**_***t*Bu** and its Pluronic formulation, respectively. In both cases, **ZnPc** and **ZnPc-P123** exhibited a decreased activity more than other modified PSs. The phototoxic potential of all tested PSs can be ordered as follows: **PtSO**_**2**_***t*Bu-P123** > **ZnSO**_**2**_***t*Bu-P123** > **PtSO**_**2**_***t*Bu** > **ZnSO**_**2**_***t*Bu** > **ZnPc-P123** > **ZnPc** (Figure S10). It is important to note that the findings derived from CRC organoids hold significant translational promise. Research indicates that three-dimensional cell clusters specific to organs, whether derived from hiPSC or resembling cancer organoids, exhibit a structural organization akin to in vivo cell sorting along with the spatial restriction and distribution of cells. This characteristic makes them an excellent model for replicating human cancer features and the diversity of cancer cells.^66^ Additionally, stem cell-derived CRC organoids emerge as a promising alternative, offering advantages such as circumventing the requirement for primary resections, which are frequently rare.^67^ It is also worth noting that although the U.S. Federal Drug Administration (FDA) traditionally requires all drugs to be tested on animals before human clinical trials, in December 2022, it amended this requirement. Now drugs that have been thoroughly tested on non-animal models, such as organoids, can proceed to clinical testing under certain criteria.

## In Vivo Experiments

### Real-Time In Vivo Imaging

**Figure 9:**
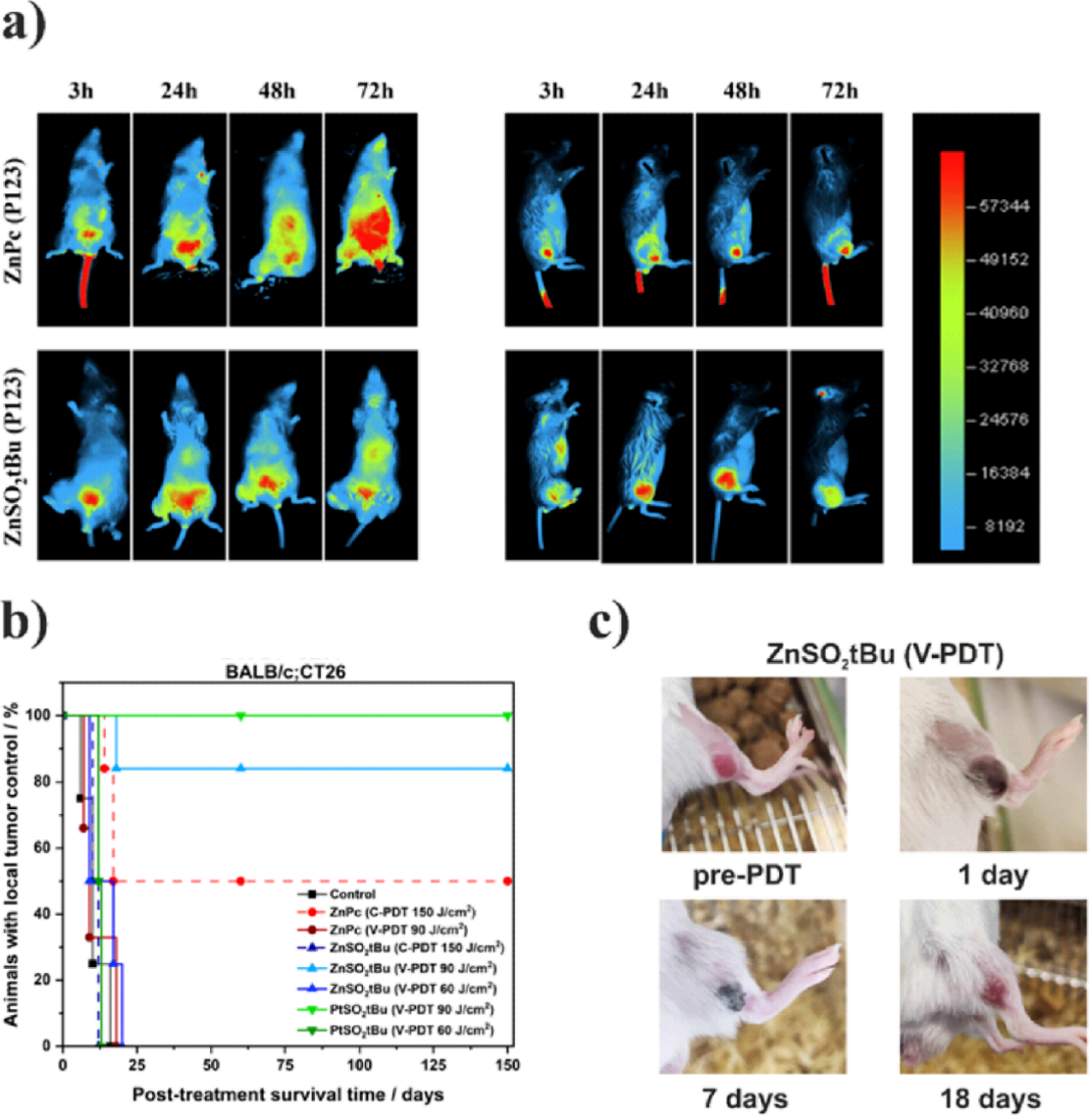
(a) Whole-body fluorescence imaging of BALB/c mice bearing CT26 tumor in the right leg, collected after 3, 24, 48, and 72 h after the i.v. administration of 1.5 mg/kg of **ZnPc-P123** and **ZnSO**_**2**_***t*Bu-P123** with excitation at 680 nm (excitation range: 650–690 nm) and fluorescence emission at 750 nm (detection range: 710–755 nm), (b) antitumor efficacy of **ZnPc-P123**, **ZnSO**_**2**_***t*Bu-P123,** and **PtSO**_**2**_***t*Bu-P123** formulated in Pluronic P123 micelles against CT26 tumors in BALB/c mice model at DLI = 15 min (V-PDT) and 24 h (C-PDT) and (c) observations of changes in tumor before, 1 day after, 7 days after and 18 days after PDT (with **ZnSO**_**2**_***t*Bu-P123**) showing the development of local necrosis, formation of a scab and a healthy tissue regeneration.

In vivo fluorescence imaging was used to follow the successful accumulation of **ZnPc-P123** and **ZnSO**_**2**_***t*Bu-P123** in tumor tissues. **PtSO**_**2**_***t*Bu**-**P123** was not utilized for real-time in vivo experiments due to its low fluorescence quantum yield, preventing observable changes in mice imaging both with and without an administrated PS. In vivo fluorescence imaging at different time intervals after i.v. administration of **ZnPc-P123** and **ZnSO**_**2**_***t*Bu-P123** phthalocyanines (1.5 mg/kg) was carried out on BALB/c tumor-bearing CT26 mice. Imaging was performed using phthalocyanine excitation at 680 nm and fluorescence signal detection in the range of 740–780 nm (Figure 9a). Figure 9a shows representative images of mice with tumors in the right leg after i.v. injection of **ZnPc-P123** or **ZnSO**_**2**_***t*Bu-P123** (left and right panels, respectively) delivered in P123 micelles. The fluorescence images show that **ZnPc-P123** accumulates up to 72 h after injection. In mice with PS-treated tumors, **ZnSO**_**2**_***t*Bu-P123** accumulation in the tumor was evident 3 h after i.v. administration and reached a maximum after 24 h and persisted until 48 h. Accumulation in the tumor remained high even at 72 h after injection. A small signal in the peritoneum was evident at each imaging time point. The accumulation of **ZnPc-P123** and **ZnSO**_**2**_***t*Bu-P123** takes the same amount of time, but in the case of **ZnPc-P123**, significant fluorescence from the mouse tail vein is visible in all images over time, which is not observed with **ZnSO**_**2**_***t*Bu-P123**. Furthermore, the fluorescence intensity at the tumor site in mice was remarkably constant.

#### PDT Treatment

The biological mechanism and resulting efficacy of PDT can be tuned by modulation of the drug-light interval (DLI). In this regard, we investigated **ZnSO**_**2**_***t*Bu-P123**, **PtSO**_**2**_***t*Bu-P123**, and **ZnPc-P123** in two different treatment regimens: V-PDT (DLI = 15 min) and C-PDT (DLI = 24 h) for the treatment of colon tumor (CT26)-bearing BALB/c mice. All tested compounds were formulated in Pluronic-P123 micelles and injected i.v. in the tail vein at a dose of 1.5 mg/kg body weight (BW). Depending on the chosen protocol after appropriate DLI, the tumor area was exposed to 60 J/cm^2^ (suboptimal dose, V-PDT), 90 J/cm^2^ (V-PDT), or 150 J/cm^2^ (C-PDT) with 652 nm laser illumination. During all protocols, an appropriate irradiation margin was maintained to ensure the effectiveness of therapy. The evaluation of phthalocyanines effectiveness in PDT was conducted by measurement of tumor growth kinetics in untreated animals and PDT-treated animals. Therapeutic efficacy was analyzed in terms of mice post-treatment survival time, and the results obtained for different protocols are presented as Kaplan–Meier plots in Figure 9b. The kinetics of average tumor growth in animals are shown in Figure S11. The experiments were conducted in accordance with the protocol (permission no 242/2022) considering the number of animals planned for procedures approved by the Local Institutional Animal Care and Use Committee (IACUC), Krakow, Poland.

### V-PDT

The highest effectiveness was achieved for **PtSO**_**2**_***t*Bu-P123**-V-PDT with a 90 J/cm^2^ light dose (100% of long-term cures) and **ZnSO**_**2**_***t*Bu-P123** in the same protocol (84% long-term cures). We observed differences in acute biological responses to PDT, such as edema and erythema, in protocols employing different PSs. For **ZnSO**_**2**_***t*Bu-P123**, under the application of a light dose of 90 J/cm^2^, a moderate immune response was observed. After laser exposure, erythema manifested in the tumor region, followed by local necrosis and edema after 24 h. Subsequently, a scab formation process commenced after 48 h, culminating in a fully developed scab with slight reddening by 72 h. The scab abated within an average span of 2 weeks, leaving behind a minor scar (Figure 9c). However, with **PtSO**_**2**_***t*Bu-P123**, immediate erythema was more pronounced following treatment, and extensive necrosis was initiated after 48 h, reaching its peak after 72 h. This implies that for **ZnSO**_**2**_***t*Bu-P123**, a 90 J/cm^2^ dose is required to achieve the desired efficacy. For the platinum derivative, where singlet oxygen production is higher at the same light dose, resulting in a conspicuously heightened response of the animal organism, a therapeutic window exists, wherein the compound continues to be effective. An exploratory attempt at **PtSO**_**2**_***t*Bu-P123**-V-PDT therapy with a light dose of 72 J/cm^2^ resulted in tumor disappearance, with a similar response observed for the zinc derivative at a 90 J/cm^2^ light dose (Figure 9d). While this outcome was not incorporated into the survival analysis due to its lack of statistical significance, it lends support to the proposition that manipulating the light dose and potentially the compound dosage with **PtSO**_**2**_***t*Bu**-V-PDT is feasible while preserving efficacy and avoiding an excessive immune response.

Upon the reduction of the light dose to 60 J/cm^2^, both compounds employed in V-PDT exhibited negligible therapeutic effects with no distinctions between the untreated control group and the treated animals. No long-term cures were observed for **ZnPc-P123** in the V-PDT protocol at a light dose of 90 J/cm^2^.

### C-PDT

For the C-PDT protocol, **ZnPc-P123** resulted in 50% of long-term cures, while **ZnSO**_**2**_***t*Bu** exhibited no effects, and the extension of survival time did not attain statistical significance compared with the untreated controls. As **ZnSO**_**2**_***t*Bu-P123** did not give any positive results in the C-PDT approach, the attempt to examine the effectiveness of **PtSO**_**2**_***t*Bu-P123** in the same protocol was abandoned. For **ZnPc-P123**, 24 h post-treatment, the tumor area became pale, suggesting a potential influx of immune cells into the tumor (Figure S12). Subsequently, within the subsequent 48 h, scab formation ensued, dissipating within less than 2 weeks. The data from tumor growth observations for all cases has been collected in Table S11.

It is widely known that vascular versus cellular targeting depends critically on the relative distribution of the PS in each compartment and its pharmacokinetic properties and can be effectively controlled not only by DLI but also by adjusting the molecular structure of the PS. Therefore, modification of the **ZnPc-P123** structure obtaining **ZnSO**_**2**_***t*Bu-P123** and **PtSO**_**2**_***t*Bu-P123** allowed phthalocyanine to be used in the V-PDT approach. This vascular-targeted treatment is possible as **ZnSO**_**2**_***t*Bu-P123** and **PtSO**_**2**_***t*Bu-P123** bind efficiently to serum albumin and remain in the bloodstream, which makes them easy to target in tumor vasculature. To the best of our knowledge, this is the best result with such high rates of long-term cures, for a phthalocyanine-based PS in V-PDT ever reported in the literature.

## Summary and Conclusions

We have designed and synthesized two phthalocyanines substituted with *tert*-butylsulfonyl groups, one metalated with a zinc(II) ion (referred to as **ZnSO**_**2**_***t*Bu**) and the other one by a platinum(II) ion (referred to as **PtSO**_**2**_***t*Bu**). We then compared their spectroscopic, photophysical, and photochemical properties to those of unsubstituted **ZnPc**. The results revealed that both **ZnSO**_**2**_***t*Bu** and **PtSO**_**2**_***t*Bu** are notably stable under light exposure. **PtSO**_**2**_***t*Bu** exhibited a significant improvement in the production of singlet oxygen (with a quantum yield of ∼90%, compared to ∼50% for **ZnPc** and **ZnSO**_**2**_***t*Bu**), while **ZnSO**_**2**_***t*Bu** demonstrated an enhanced ability to generate Type I photoproducts, most notably hydroxyl radicals.

By substituting phthalocyanine at four positions with a *tert*-butylsulfonyl group, we improved several pharmacological factors, as well as its uptake by cells and its overall effectiveness in in vitro PDT. When designing PSs for clinical use, it is crucial to consider their solubility in biocompatible and safe carriers. Balancing these factors with high lipophilicity can be challenging, which has prompted various efforts to create PSs that are more hydrophilic while still capable of targeting tumors. For instance, a liposomal formulation of unsubstituted **ZnPc** (CGP55847) proved to be a promising candidate for clinical PDT.

The outcomes of the interaction between phthalocyanines and plasma proteins validate the formation of a stable complex with serum albumin for both **ZnSO**_**2**_***t*Bu** and **PtSO**_**2**_***t*Bu**. This knowledge is of utmost significance as in V-PDT, we are essentially exciting the formed Pc-albumin complex rather than just the PS alone. Previous studies on bacteriochlorophyll derivatives, specifically WST11,^68^ have indicated an enhanced production of ROS species upon irradiation in the presence of the Pc-BSA complex. This underscores the critical role of understanding complex formation in influencing the outcomes of PDT and has implications for optimizing therapeutic strategies in V-PDT.

**ZnSO**_**2**_***t*Bu-P123** and **PtSO**_**2**_***t*Bu-P123** demonstrate comparable phototherapeutic characteristics in vitro, prompting the initiation of in vivo experiments involving both phthalocyanine derivatives. Previous studies of the effect of micellization in Pluronic P123 on the pharmacokinetics and bioavailability of the administrative drug^69^ showed no significant effect on these parameters for paclitaxel. In addition, the use of P123 as a carrier for phthalocyanines allows working with nontoxic solvents, which would be impossible with phthalocyanines alone due to their tendency to aggregate in aqueous solutions. In light of these results and our own in vitro studies, which showed that the phthalocyanines encapsulated in P123 exhibited a superior phototherapeutic effect for the A549, 2H11, LLC CT26, and MCF-7 cell lines, we decided to continue in CRC organoids and in vivo studies for **ZnSO**_**2**_***t*Bu-P123** and **PtSO**_**2**_***t*Bu-P123** only. This aspect is crucial in the context of adhering to the 3Rs principles,^70^ which mandate humane and responsible in vivo research and minimizing the number of animals used to the minimum necessary.

Upon analyzing the optical and photophysical characteristics of **ZnSO_2_*t*Bu** and **PtSO_2_*t*Bu** in both encapsulated and nonencapsulated forms, it becomes clear that micellization reduces aggregation, enhances stability, and boosts lethality across different cell lines. This phenomenon may be associated with an augmented generation of ROS due to an extended action time against cells in in vitro, organoids, and in vivo experiments.

**ZnSO**_**2**_***t*Bu** exhibited a higher capability for generating hydroxyl radicals in comparison to singlet oxygen. Moreover, it exhibited efficient fluorescence, enabling the non-invasive monitoring of its accumulation in tumors and kinetics post in vivo administration through fluorescence imaging. Intriguingly, **ZnSO**_**2**_***t*Bu** displayed impressive efficacy in PDT, impeding tumor growth for more than five months with a single light dose of 90 J/cm^2^, resulting in complete cure in tested mice. This heightened photodynamic effectiveness can be ascribed to its uncomplicated and safe composition, outstanding in vitro phototoxicity, and selective tumor accumulation. In a BALB/c mouse model with CT26 tumors, V-PDT utilizing **ZnSO**_**2**_***t*Bu-P123** conferred a significant survival advantage, leading to 84% long-term cures.

**PtSO**_**2**_***t*Bu-P123** demonstrated a greater ability to generate singlet oxygen in comparison to hydroxyl radicals. Due to its low fluorescence, conducting real-time in vivo experiments proved unfeasible. Nevertheless, **PtSO**_**2**_***t*Bu-P123** exhibited impressive efficacy in PDT, hindering tumor growth not only with a light dose of 90 J/cm^2^ but also with a dose of 72 J/cm^2^, resulting in a 100% cure rate. Consequently, **PtSO**_**2**_***t*Bu-P123** provides increased flexibility in adjusting the light dosage to achieve the desired therapeutic outcomes. This notable outcome positions **ZnSO**_**2**_***t*Bu-P123** and **PtSO**_**2**_***t*Bu-P123** as highly promising phthalocyanine-based PSs for V-PDT in colorectal cancer. It is noteworthy that the phthalocyanines we designed, prepared, characterized, and tested are the first compounds from this group to demonstrate such high effectiveness in V-PDT. So far, phthalocyanines have been used in PDT protocols targeting cancer cells with DLI of 24 h. We believe that our work will change the perspective on phthalocyanines as PSs and expand their potential use also for V-PDT.

## Experimental Section

### Materials and Methods

#### Synthesis

Infrared spectra (IR) were captured using a Bio-Rad FTS 175C Fourier transform infrared (FTIR) spectrophotometer (Figures S15 and S18). Mass spectra were recorded on a matrix-assisted laser desorption ionization (MALDI) BRUKER Microflex LT using Dithranol (DIT) or 2,5-dihydroxybenzoic acid (DBH) as the matrix (Figures S13 and S16). NMR spectra were recorded in deuterated chloroform (CDCl_3_) on a Varian 500 MHz spectrometer (Figures S14 and S17).

#### Synthesis of 3-*tert*-Butylsulfanyl Phthalonitrile

A mixture of 3-nitrophthalonitrile (5 g, 28.9 mmol), *tert*-butylthiol (2.6 g, 28.9 mmol), and dry potassium carbonate (20 g, 0.14 mol) in DMF (30 mL) was stirred at room temperature under an argon atmosphere for 24 h. The reaction mixture was poured then into 250 mL of water. The solid obtained was gathered through filtration and subsequently rinsed with water. After drying in vacuum, the crude product was recrystallized from ethanol. Yield: 75% (4.8 g). MALDI-TOF-MS (matrix DIT): *m*/*z* 217.70 [MH]^+^ calculated for C_12_H_12_N_2_S: 216.30. ^1^H NMR (500 MHz, CDCl_3_): δ, ppm 1.38 (s, 9H), 7.69 (t, 1H), 7.80 (d, 1H), 7.92 (d, 1H). ^13^C NMR (125 MHz, CDCl_3_): δ, ppm 142.4, 139.6, 133.3, 132.3, 123.8, 117.4, 115.3, 114.8, 110.0, 109.9, 50.2, 31.0. FT-IR (ν, cm^–1^): 2966, 2929, 2862, 2237, 1567, 1471, 1457, 1444, 1421, 1390, 1366, 1163, 1139, 934, 856, 806, 742.

#### Synthesis of 3-*tert*-Butylsulfonyl Phthalonitrile

3-*tert*-Butylsulfanyl phthalonitrile (3 g, 13.86 mmol) was stirred in acetic acid (60 mL) at 90 °C, and then a 33% solution of H_2_O_2_ (80 mL) was added over 4 h. The stirring at 90 °C continued overnight, then water (250 mL) was added to the cooled reaction mixture. The white precipitate was collected by filtration, washed with water, and crystallized from ethanol. Yield: 92% (3.2 g). MALDI-TOF-MS (matrix DHB): *m*/*z* 248.89 [M]^+^ calculated for C_12_H_12_N_2_O_2_S: 248.30. ^1^H NMR (500 MHz, CDCl_3_): δ, ppm 1.45 (s, 9H), 7.95 (t, 1H), 8.09 (d, 1H), 8.31 (d, 1H). ^13^C NMR (125 MHz, CDCl3): δ, ppm 140.2, 137.4, 136.6, 132.9, 119.9, 117.4, 114.3, 113.1, 110.0, 109.9, 62.9, 23.7. FT-IR (ν, cm^–1^): 2981, 2933, 2233, 1580, 1562, 1475, 1434, 1396, 1372, 1301, 1204, 1191, 1150, 1109, 941, 849, 806, 782.

### General Procedure for the Synthesis of **ZnSO_2_*t*Bu** and **PtSO_2_*t*Bu**

3-*tert*-Butylsulfonylphthalonitrile (250 mg, 0.90 mmol) was heated at 130 °C in a mixture of DMF/*o*-dichlorobenzene (1:3) under argon for 12 h in the presence of the corresponding metallic salt (Zn(OAc)_2_ or PtCl_2_) (0.45 mmol). The solvents were removed under reduced pressure. The green-blue waxy solid was extracted with CH_2_Cl_2_ and washed with water. Phthalocyanines are then purified by column chromatography on silica gel using a dichloromethane/ethanol mixture of dichloromethane–ethanol (100:1).

### ZnSO_2_*t*Bu

Yield: 38% (103 mg). MALDI-TOF-MS (matrix DIT): *m*/*z* 1058.09 [M]^+^ calculated for C_48_H_48_N_8_O_8_S_4_Zn: 1058.58, *m*/*z* 939.02 [M-SO_2_*t*Bu]^+^. ^1^H NMR (500 MHz, CDCl_3_): δ, ppm 1.28 (s, 12H), 1.44–1.72 (m, 24H), 7.90–8.58 (m, 6H), 8.82–8.92 (d, 2H), 9.38 (s, 1H), 9.79 (d, 2H), 10.26 (s, 1H). FT-IR (ν, cm^–1^): 2921, 2847, 1554, 1433, 1296, 1180, 1088, 818, 750.

### PtSO_2_*t*Bu

Yield: 35% (102 mg), MALDI-TOF-MS (matrix DIT): *m*/*z* 1188.82 [M]^+^ calculated for C_48_H_48_N_8_O_8_PtS_4_: 1188.28. ^1^H NMR (500 MHz, CDCl_3_): δ, ppm 0.25 (s, 9H), 1.72–1.92 (m, 27H), 8.56 (m, 4H), 9.07 (m, 4H), 10.52 (m, 4H). FT-IR (ν, cm^–1^): 2931, 2861, 1614, 1517, 1461, 1398, 1306, 1269, 1143, 1113, 1091, 1049, 938, 838, 756, 712.

### Triplet State Lifetime Measurements

The triplet state lifetimes were measured with the laser flash photolysis spectrometer at 20 °C. All phthalocyanine solutions were freshly prepared before the measurements. To conduct experiments in an oxygen-free environment, the solutions underwent argon purging until a stable decay rate was achieved and they remained under argon throughout the measurements. The curves were registered after the laser excitation (λ_ex_ = 355 nm).

### Detection of ROS in Solution

A fluorescent probe, 2-[6-(4-aminophenoxy)-3-oxo-9-yl] benzoic acid (APF), was used for the detection of ROS due to its selectivity to the hydroxyl radical. The measurement was carried out using 96-well plates. Solutions of PSs in THF:PBS solvent (1:99) with TRITON X-100 were prepared so that their final concentration in the well was 3 μM. The corresponding fluorescent probe was then introduced so that its final concentration in the well was 15 μM for the APF probe. Solutions of the tested PSs were illuminated with a 635 ± 20 nm laser diode at increasing time intervals. The irradiance to which the test compounds were exposed was 17 mW/cm^2^ and was monitored using a hand-held NOVA II laser power and energy meter from OPHIR. During the irradiation of the samples, the increase in the fluorescence signal intensity of the probes used was monitored. An excitation wavelength of 490 nm and an emission wavelength of 515 nm were used for the ·OH selective probe.

### *n*-Octanol/PBS Partition Coefficients

The partition coefficients of *n*-octanol/PBS phthalocyanines were determined based on the shake-flask method. A small amount of **ZnSO**_**2**_***t*Bu** and **PtSO**_**2**_***t*Bu** was dissolved in *n*-octanol saturated with PBS buffer (5 mL) and then sonicated until complete dissolution. PBS buffer saturated with *n*-octanol (5 mL) was then introduced into the system, and the whole thing was resonified. The phase mixture was shaken using a vortex shaker for about 30 min, followed by sonication for 15 min at 40 °C. Later, the solution was centrifuged at 3700 rpm for 5 min. The next step was to take a volume equal to 0.02 mL from each of the phases obtained and prepare 0.5% solutions of n-octanol or PBS in DMSO. A series of solutions of phthalocyanines in DMSO containing 0.5% *n*-octanol or PBS buffer in the concentration range of *c* = 0.1 μM–6.25 nM were also prepared. All samples were sonified for 15 min at 40 °C. The fluorescence intensity of all samples was measured using a Fluorolog-3 Spectrometer (Horiba Jobin-Yvon). The calibration curve prepared allowed the concentration of substituted phthalocyanines to be determined, and then the log *P*_OW_ partition coefficient was determined based on eq 2:
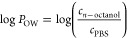
2

### Interaction with BSA Plasma Proteins

The interaction of phthalocyanines with plasma proteins was studied by quenching the fluorescence of the tryptophan residue of BSA in the presence of increasing concentrations of PS. A 1 μM solution of BSA in PBS at pH = 7.4 was prepared and then titrated (0.5 μL) with 500 μM phthalocyanine solutions in THF. After each addition of PS, the fluorescence spectrum was recorded in the wavelength range of 300–420 nm and at the excitation wavelength of 295 nm. The slit width was 5 nm, and the scanning speed was 700 nm/min. At the same time, electron absorption spectra were measured in the wavelength range of 200–350 nm and at a scan rate of 960 nm/min. Measurements of UV–Vis spectra were carried out to eliminate the internal filter effect created by the absorption of excitation radiation and emitted by compounds of the phthalocyanine group. This phenomenon contributes to a decrease in fluorescence intensity, so a correction of the fluorescence intensity value by formula 3 was included:
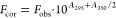
3where *F*_cor_—the fluorescence intensity after the correction taken into account, *F*_obs_—the observed fluorescence intensity, and *A*_295_ and *A*_350_—the absorbance intensities of the analyzed sample successively at the wavelength of the excitation (295 nm) and emitted radiation (350 nm). Fluorescence spectra were measured using a PerkinElmer LS55 fluorescence spectrometer, while a PerkinElmer UV–Vis Lambda 25 spectrometer was used to record electronic absorption spectra.

### Preparation of PS-Loaded Polymeric Micelles

PSs were incorporated into the poloxamers by a thin-film method. At about 50 °C, the solid poloxamer melted. Samples of phthalocyanines and Pluronic P123 were codissolved in an organic solvent (THF). The solvent was then removed by rotary evaporation during which a thin film was formed. The film was moistened with phosphate-buffered saline (PBS). The solution was centrifuged using a ThermoFisher centrifuge, Megafuge 8/8R, 8000 rpm for 10 min.

### Phototoxicity and Cell Survival Assay

An Alamar Blue (resazurin-based) test was carried out to evaluate cell viability and determine cell toxicity after the photodynamic effect with phthalocyanines. The cells were incubated with a PS solution (20 μM) for 18–20 h in the dark. After this time, the PS solution was removed, and the cells were washed twice with a PBS solution containing Ca^2+^ and Mg^2+^. Then, cells were irradiated with red light at various doses (0–15 J/cm^2^). Following the rinse, a fresh medium containing FBS and antibiotics was added to each well, and the cells were placed back into an incubator for another 24 h. Subsequently, a viability assessment was conducted. A 10% solution of resazurin was added to each well, and the microplates were incubated for 3 h. The fluorescence emitted at a test wavelength of 590 nm (excitation at 560 nm) was then measured using an automated microplate reader (Tecan Infinite M200 Reader) in order to quantify the cells' viability.

### Studies on Organoids

Human induced pluripotent stem cells (hiPSCs) were cultured in 6-well plates precoated with Geltrex (ThermoFisher) using mTeSR1 growth medium (STEMCELL Technologies). The cells were incubated at 37 °C with 5% CO_2_. To create a model of colorectal cancer, the hiPSCs were differentiated into colonic organoids according to the previously described method.^65,67^ This method involved manipulating signaling pathways to progressively generate different cell types: definitive endoderm (DE) with CHIR99021 and activin A, hindgut endoderm (HE) using CHIR99021 and FGF4, and finally colonic organoids (CO) through supplementation with CHIR99021 + LDN19318 + EGF and B27. The entire differentiation process took approximately 40 days. Over the course of the following 6 weeks, individual colonic stem cells produced colonic organoids (COs), which were transferred to new plates at a 1:4 density every 10 days. Three days before the assay, the COs were switched to a colonic medium without CHIR.

### Cytotoxicity in the Dark

The CellTox Green Cytotoxicity Assay (Promega) was employed to measure cell viability and the harmful effects caused by PSs. Organoids were initially distributed in a 96-well microplate. Following a 3 day period, they were exposed to PSs in a growth medium with concentrations ranging from 0 to 5 mg/kg, all in the absence of light. Subsequently, the solution was extracted from each well, the cells were washed with PBS, and fresh culture medium was added back into each well. The cells were subsequently placed back into the incubator for 24 h. The CellTox Green Cytotoxicity Assay was utilized to perform the assessment, and the resulting fluorescence was quantified using an automated microplate reader (Tecan Infinite M200 Reader).

### Photodynamic Effect

After analyzing the results of cytotoxicity, a safe concentration of PS (2 mg/kg BW) was selected. The organoids were exposed to the PS solution in the culture medium for 24 h in the dark. Subsequently, the organoids were washed with PBS and subjected to irradiation using a 635 ± 20 nm LED system at various time intervals. Following this, the organoids were rinsed with fresh medium and returned to the incubator for another 24 h. The viability of the organoids was evaluated in separate experiments conducted 24 h after irradiation, utilizing the CellTox Green Cytotoxicity Assay.

### Life/Dead Staining

The organoids were kept in the dark and exposed to investigated PSs for 24 h. Following incubation, the organoids were washed with PBS and subjected to irradiation using an LED system operating at a wavelength of 635 ± 20 nm. Subsequently, the cells were rinsed with fresh medium and returned to the incubator for another 24 h. To determine the viability of the organoids, a staining technique involving Hoechst33342 (10 μg/mL) and propidium iodide (1 μg/mL) was employed to distinguish between live (blue fluorescence) and dead cells (red fluorescence). Selected organoids, both treated and untreated, were washed with HBSS. Then, the organoids were stained with Hoechst33342 and PI to evaluate the proportions of live and dead cells. Afterward, they were washed twice with HBSS and made ready for visualization. Using a Zeiss LSM 880 confocal microscope equipped with a 40× immersion objective, the samples were imaged in z-stack mode. The resulting images were recorded and subsequently analyzed using Zeiss ZEN software.

### Real-Time Whole-Body Imaging

When the tumors reached ca. 0.5 cm in diameter, the mice were injected i.v. with **ZnPc-P123** or **ZnSO**_**2**_***t*Bu-P123** with a dose of 1.5 mg/kg body weight, and whole-body imaging using the Newton 7.0 Imaging System (Viber) was performed immediately following and up to 48 h after the injection. The procedure was carried out under inhalation anesthesia with 3–4% Isoflurane (Aerrane, Baxter, Poland) maintained throughout the entire experiment. Fluorescence images of BALB/c mice were collected using λ_ex_ = 680 and λ_em_ = 740–780 nm to illustrate the specific signal for phthalocyanine.

### PDT

Mice were randomly assigned to experimental groups (*n* = 5–6). The CT26 cells (0.35 × 10^6^ in PBS suspension/100 μL) were implanted subcutaneously into the right thigh of BALB/c mice. The i.v. administration of PSs (**ZnPc-P123**, **ZnSO**_**2**_***t*Bu-P123**, or **PtSO**_**2**_***t*Bu-P123**) was done when the tumor attained a diameter of 4–5 mm, which usually took about 7–10 days after inoculation. Tumor irradiation was performed at DLI = 15 min or DLI = 24 h using a laser (Omicron laser model PDT652.2-500) with a wavelength of 652 nm and radiation exposure in the range of 60 J/cm^2^ (15 min DLI) and 100 J/cm^2^ (24 h DLI). The illuminated region, with a consistent diameter of 1.3 cm, was maintained throughout the irradiation process. During the experiment, the well-being of animals was monitored (tumor size, weight, behavior). When the tumors escaped local tumor control (diameter over 10 mm), the mice were euthanized. Survival curves were estimated using Kaplan–Meier analysis.

## Supporting Information Available

Instruments and general procedures for optical properties, photodegradation tests, determination of singlet oxygen quantum yields, characterization of PS-loaded polymeric micelles, cell culture, PS accumulation in organoids, animal model and statistical analysis, electronic absorption and fluorescence spectra, distribution of normalized weighted differences function, time-resolved fluorescence decay profile, singlet oxygen quantum yields, triplet state decays and influence of oxygen on the decay, quenching of ^1^O_2_ phosphorescence after purging the DMF solutions with argon, emission spectra phthalocyanines in different phases (*n*-octanol, PBS) and calibration curves in PBS, fluorescence quenching of BSA, dynamic light scattering, kinetic curves of tumor growth, observation of changes in the tumor, observations of changes in tumors with measurements of tumor sizes, phthalocyanine characterization spectra (PDF)

## Abbreviations

·OH: hydroxyl radical
^1^Δ_g_: singlet oxygen
2H11: murine endothelial/vascular epithelium
A549: human lung adenocarcinoma
AMD: age-related macular degeneration
APF: aminophenyl fluorescein
BSA: bovine serum albumin
BW: body weight
DLI: drug-to light interval
C-PDT: cellular-targeted photodynamic therapy
CRCs: human colorectal cancers
CT26: murine colorectal carcinoma
DAPI: 2-(4-amidinophenyl)-1*H*-indole-6-carboxamidine
DBH: 2,5-dihydroxybenzoic acid
DIT: dithranol
DLS: dynamic light scattering
DMEM: Dulbecco’s Modified Eagle Medium
DMF: *N,N*-dimethylformamide
DMSO: dimethyl sulfoxide
DPBF: 1,3-diphenyl-2-benzofuran
H_2_O_2_: hydrogen peroxide
HBSS: Hanks' balanced salt solution
hiPSC: human-induced pluripotent stem cells
i.v.: intravenous
IR: infrared spectroscopy
IRF: instrument response function
*K*_b_: binding constant
*k*_qBSA_: molecular quenching constant for albumin
*k*_q_: quenching rate constant
*K*_sv_: Stern–Volmer constant
LDL: low-density lipoprotein
LLC: Lewis lung carcinoma
LLD_50_: lethal light dose that caused 50% mortality
MALDI: matrix-assisted laser desorption ionization
MCF-7: human breast cancer cells
MRP: multidrug resistance protein
NIR: near-infrared radiation
NMR: nuclear magnetic resonance
O_2_^·–^: superoxide ion
*o*-DCB: 1,2-dichlorobenzene
PBS: phosphate-buffered saline
PDT: photodynamic therapy
PEG: polyethylene glycol
PGP: P-glycoprotein
*P*_ow_: partition coefficient
PPG: polypropylene glycol
PS: photosensitizer
Pt(OAc)_2_: platinum(II) acetate
PtSO_2_*t*Bu: platinum(II) 1,8,18,22-tetrakis(*tert*-butylsulfonyl)phthalocyanine
ROS: reactive oxygen species
TCSPC: time-correlated single photon counting
THF: tetrahydrofuran
τ_T_: triplet state lifetimes
V-PDT: vascular-targeted photodynamic therapy
Zn(OAc)_2_: zinc(II) acetate
ZnPc: zinc(II) phthalocyanine
ZnSO_2_*t*Bu: zinc(II) 1,8,18,22-tetrakis(*tert*-butylsulfonyl)phthalocyanine
Δλ_emSt_: Stokes shift
ε: molar absorption coefficient
λ_em_: maximum emission wavelength
λ_max_: maximum absorption wavelength
τ_F_: fluorescence lifetime
Φ_F_: fluorescence quantum yield
Φ_Δ_: singlet oxygen quantum yield
