# Supplementary material for: Synthesis, Photo-Characterizations, and Pre-Clinical Studies on Advanced Cellular and Animal Models of Zinc(II) and Platinum(II) Sulfonyl-Substituted Phthalocyanines for Enhanced Vascular-Targeted Photodynamic Therapy
Source: ACS Appl Mater Interfaces. 2024 Sep 6;16(37):48937–54. doi: 10.1021/acsami.4c04138 (PMC11420872; doi:10.1021/acsami.4c04138)
Supplement: Supplementary file 1 — am4c04138_si_001.pdf [file am4c04138_si_001.pdf]

Supporting Information for:

Synthesis, Photo-Characterizations and Pre-Clinical Studies on Advanced Cellular and Animal Models of Zinc(II) and Platinum(II) Sulfonyl-Substituted Phthalocyanines for Enhanced Vascular-Targeted Photodynamic Therapy

Paweł Repetowski,[a,b] *Marta Warszyńska,*[a,b] *Anna Kostecka,*[a] *Barbara Pucelik,*[c,d] *Agata Barzowska,*[c,d] *Atefeh Emami,*[f] *Ümit İşci,*[e] *Fabienne Dumoulin,*[f,*] *and Janusz M. Dąbrowski* [a,*]

[a]Faculty of Chemistry, Jagiellonian University, 30-387 Kraków, Poland

[b]Doctoral School of Exact and Natural Sciences, Jagiellonian University, 30-348 Kraków, Poland

[c]Małopolska Centre of Biotechnology, Jagiellonian University, 30-387 Kraków, Poland

[d]Łukasiewicz Research Network – Kraków Institute of Technology, 30-418, Kraków, Poland

[e]Marmara University, Faculty of Technology, Department of Metallurgical & Materials Engineering, 34722, Istanbul, Türkiye

[f]Acıbadem Mehmet Ali Aydınlar University, Faculty of Engineering and Natural Sciences, Department of Biomedical Engineering, 34752, Ataşehir, Istanbul, Türkiye

* Correspondence should be sent to Janusz M. Dąbrowski, jdabrows@chemia.uj.edu.pl, +48126632293, +48126340515 (fax)

Or Fabienne Dumoulin, e-mail: fabienne.dumoulin@acibadem.edu.tr

Table of Contents

INSTRUMENTS AND GENERAL PROCEDURES…………………………………………..….**3**

Figure S1 Electronic absorption and fluorescence spectra…………….………..………..…**7**

Figure S2 Distribution of normalized weighted differences in phthalocyanine compounds and Time-resolved fluorescence decay profile…..………………..……………..………………**8**

Figure S3 Singlet oxygen quantum yields……...……….……………………….……………**9**

Figure S4 Triplet state decays and influence of oxygen on the decay…………………………**9**

Figure S5 Quenching of $^1O_2$ phosphorescence……………….…………………………………**10**

Figure S6 Emission spectra for logP$_{OW}$ determination…..……………………..……...……..**11**

Figure S7 Fluorescence quenching of BSA…………………………..…..………………..**12**

Figure S8 Dynamic Light Scattering………………………...…………………………….**13**

Figure S9 Photodynamic effect against A549, LLC, MCF-7 and 2H11 cells…………….….**14**

Figure S10 The red pixels quantification after photodynamic effect on organoids…………**14**

Figure S11 Kinetic curves of tumor growth...………………..……...…………….. ……..**15**

Figure S12 Observations of changes in tumor…..…………………………………………**16**

Table S1 Observations of changes in tumors with measurements of tumor sizes……………**17**

Characterization spectra **for ZnSO$_2$tBu**…………………….……………………………..**18**

Characterization spectra **for PtSO$_2$tBu**…………………….……………………………...**19**

INSTRUMENTS AND GENERAL PROCEDURES

Optical properties. Electronic absorption measurements were made using a Hewlett Packard HP8453 UV/Vis spectrophotometer, recording spectra in the 190-850 nm range. Solutions representing the test compounds dissolved in a suitable solvent were placed in quartz cuvettes with an optical path of 1 cm. The molar absorption coefficients were determined from Beer's law. Fluorescence spectra were recorded using a Perkin Elmer LS 55 Fluorescence Spectrometer, with a slit width of 8 nm and a scan rate of 100 nm/min. The fluorescence spectra were recorded at an excitation radiation wavelength of 592 nm. The fluorescence lifetimes of the compounds were recorded with a Fluorolog-3 Spectrometer (Horiba Jobin-Yvon) using Time-Correlated Single Photon Counting (TCSPC). Measurements were carried out using a picosecond pulsed diode with a wavelength of 372 nm and for an excitation pulse duration of 200 ns. Fluorescence decay curves for each compound were recorded at the wavelength corresponding to its emission maximum, and the maximum number of counts per peak channel was 10000. Instrument response function (IRF) was obtained by using a standard, aqueous colloidal silica solution, LUDOX. The collected data were analyzed using Horiba Jobin-Yvon's DAS6 v6.4 program. Two-exponential models were fitted to measure fluorescence decay over time for phthalocyanines in THF, so that CHiQ values were in the range of 1.2-1.4, and residuals were symmetric about the zero axis.

Photodegradation tests. The photodegradation process of phthalocyanines was evaluated by spectrophotometric method using an Infinite M200 microplate reader from Tecan. Solutions of photosensitizers in THF:PBS (1:99) with TRITON X-100 were exposed using a 635±20 nm laser diode as a light source. The irradiance to which the test solutions were exposed was 17 mW/cm^2 and was monitored using a handheld NOVA II laser power and energy meter from OPHIR. During the irradiation of the samples, a cut-off < 550 nm filter was used to eliminate

radiation below 550 nm. During the irradiation, the absorbance value at the Q-band maximum of the photosensitizer solutions was measured after successively increasing time intervals.

Determination of singlet oxygen quantum yields (Φ_Δ). The quantum yield of singlet oxygen generation by phthalocyanines in DMF was determined by an indirect method involving chemical quenching using 1,3-diphenylisobenzofuran (DPBF) as the acceptor $^1O_2$. The values of Φ_Δ were determined by the comparative method, using the relation (1):

$$\boldsymbol{\Phi_\Delta = \Phi_{\Delta(Std)} \cdot \frac{R \cdot I_{Std}}{R_{Std} \cdot I}} \tag{1}$$

Where Φ_Δ and $\Phi_{\Delta(Std)}$ are the quantum yields of singlet oxygen generation for the sample and standard, R_{Std} and R are the photobleaching rates of the standard and phthalocyanine, respectively, and I_{Std} and I are the absorbance intensities of the standard and test compound. A commercially available zinc phthalocyanine with known singlet oxygen generation quantum yield (Φ_Δ=0.56 in DMF) was used as a standard. Working solutions were prepared, one of which was a 6 μM solution of DPBF in DMF (A ~1.3), while the other was a 5 μM solution of the corresponding phthalocyanines. The procedure consisted of irradiating the compound mixture placed in a quartz cuvette at a volume ratio of 1:1 with a xenon lamp (XBO 150) in the presence of a cut-off filter < 550. The irradiance, monitored with a handheld NOVA II laser power and energy meter from OPHIR, was ~67 mW/cm^2. The singlet oxygen generating capacity of phthalocyanines was evaluated by changing the absorbance intensity of the acceptor band maximum $^1O_2$ (414 nm) during sample irradiation. The value of singlet oxygen generation efficiency for **ZnSO$_2$tBu** and **PtSO$_2$tBu** was also determined based on stationary measurements using the single-point method. For this purpose, solutions of the standard (phenalenone) and the test substances in DMF were prepared so that their absorbances at the wavelength of the excitation radiation were equal. For the prepared solutions, singlet oxygen emission spectra were recorded at the excitation light wavelength λ_{ex}= 350 nm (absorbance ~0.20), using a cut-off filter < 715 nm. Measurements were made with a Fluorolog-3

spectrophotometer (Horiba Jobin-Yvon) equipped with a LATERAL 5509 detector cooled with liquid nitrogen for near-infrared (NIR) measurements. Considering the correction for the slight difference in absorbance at λ_{ex}, the values of Φ_Δ were determined based on the relation (2),

$$\boldsymbol{\Phi_\Delta} = \boldsymbol{\Phi_{\Delta(Std)}} \cdot \left[\frac{Area\left(\frac{T_1}{R_1}\right)}{Area\left(\frac{T_1}{R_1}\right)_{Std}}\right] \cdot \left[\frac{1-10^{A_{Std}}}{1-10^{A}}\right] \cdot \left(\frac{n^2}{n^2_{Std}}\right) \quad (2)$$

where: Φ_Δ and $\Phi_{\Delta(Std)}$ the quantum yield of singlet oxygen generation for the test sample and standard, $\mathrm{Area}\left(\frac{T_1}{R_1}\right)$ oraz $\mathrm{Area}\left(\frac{T_1}{R_1}\right)_{Std}$ the areas under the singlet oxygen phosphorescence curves analogously for the sample and standard, A and A_{Std} correspond to the absorbance intensities of the phthalocyanines and the standard at the wavelength of the excitation radiation, n and n_{Std} are the refractive indices for the solution for the test compounds and the standard, respectively. In addition, to investigate the possible contribution of the phosphorescence phenomenon of the tested compounds, analogous measurements were carried out after the solutions were blown with argon for 15 minutes.

Characterization of PS-loaded polymeric micelles. Micelle size distribution was measured by dynamic light scattering (DLS) using Malvern Zetasizer Nano ZS.

Cell culture. The following cell lines were utilized in the *in vitro* studies: human lung adenocarcinoma (A549), murine colorectal carcinoma (CT26), murine endothelial/vascular epithelium (2H11) were cultured in DMEM supplemented with 4.5 g/L glucose (BioTech). Lewis Lung Carcinoma (LLC) and human breast cancer cells (MCF-7) were cultured in RPMI1640 medium. All media were supplemented with 10% of FBS and 1% of antibiotics. Prior to the experiments, the cells were detached using trypsinization, seeded in microplates, and maintained in a humidified environment at 37°C with 5% CO_2.

PS accumulation in organoids. The organoids were subjected to a 24-hour incubation with photosensitizers solutions at a concentration of 1.5 mg/kg BW. Following this incubation period, the organoids were washed two times with PBS, and Hoechst33342 was introduced to the samples for 15 minutes. Subsequently, the samples were rinsed twice with HBSS and made ready for visualization. The imaging process was conducted using a Zeiss LSM 880 confocal microscope (Carl Zeiss, Jena, Germany) equipped with a 40× immersion objective. The resulting images were captured and analyzed utilizing Zeiss ZEN software.

Animal model.

Animal model. All experiments were carried out with approval no 242/2022 of the 2nd Local Institutional Animal Care and Use Committee (IACUC), Krakow, Poland. BALB/c mice (10−12-weeks-old males, AnimaLab sp. z.o.o) were used to perform the *in vivo* evaluation. The animals were humanely treated and supplied with food and water *ad libitum*. The animals were housed and maintained in individually ventilated cages under a 50–60% humidity, 12/12 h light/dark cycle and at 22 ± 2 °C, in SPF conditions at the Faculty of Biochemistry, Biophysics and Biotechnology, Jagiellonian University in Kraków, Poland.

The mean size of the tumors were calculated according to the following formula (3):

$$\emptyset = \sqrt[3]{a \cdot b \cdot c} \tag{3}$$

Where Ø is mean tumor diameter, a,b,c – diameters in three different dimensions.

Statistical Analysis. The STATISTICA software for biostatistics (StatSoft Inc.) was used for statistical analysis of the data. The data are expressed as means ± standard deviation or standard error of the mean (SEM) of at least three independent experiments. Statistical significance was determined by one-way or two-way ANOVA with Bonferroni post hoc test using GraphPad Prism version 5.0.0 for Windows, GraphPad Software, San Diego, California USA.

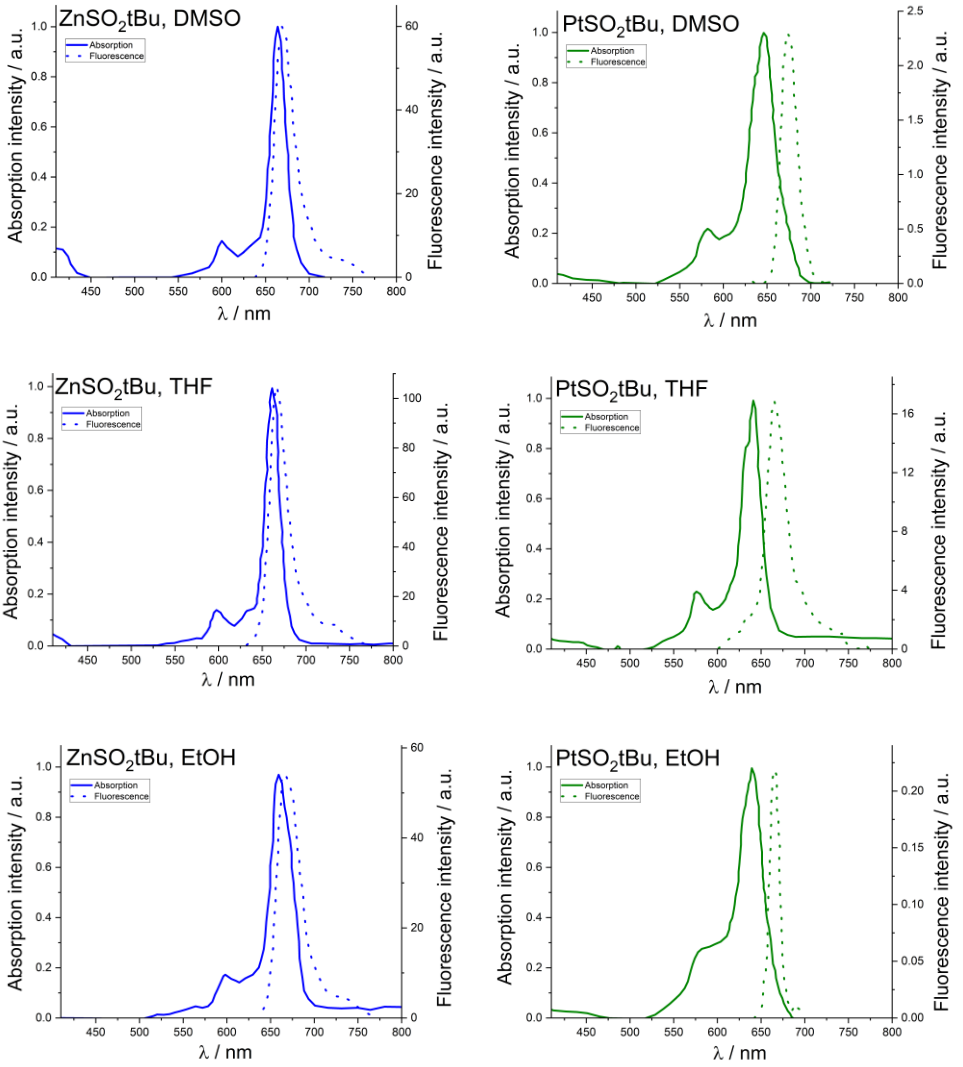

Figure S1 Electronic absorption and fluorescence spectra of **ZnSO$_2$tBu** and **PtSO$_2$tBu** measured in THF, ethanol, and DMSO at room temperature.

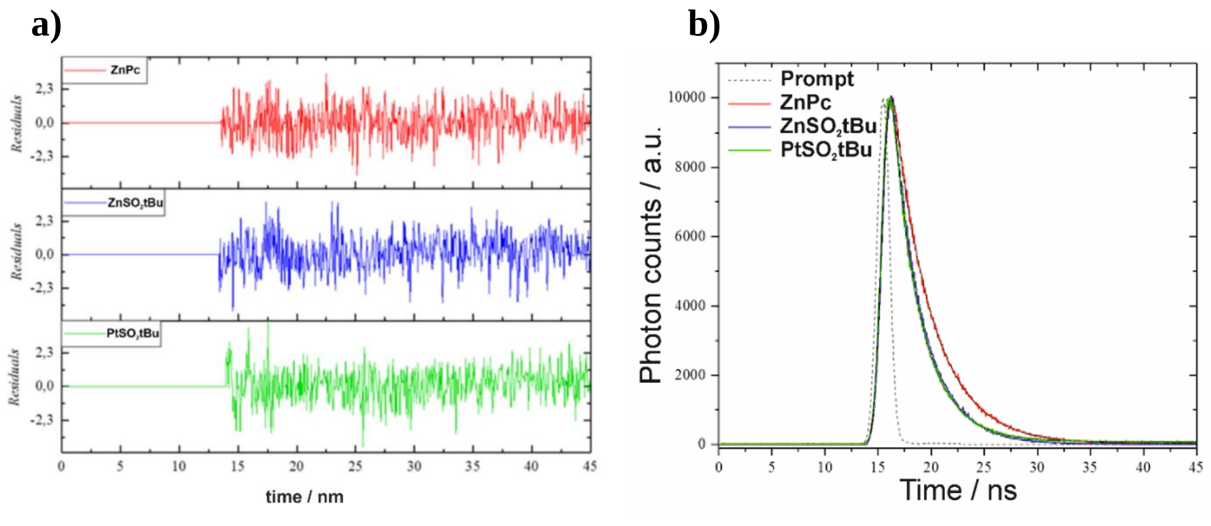

Figure S2 a) Distribution of the normalized weighted difference function for individual compounds in the phthalocyanine group. **b)** Time-resolved fluorescence decay profile for each phthalocyanine registered in THF at room temperature.

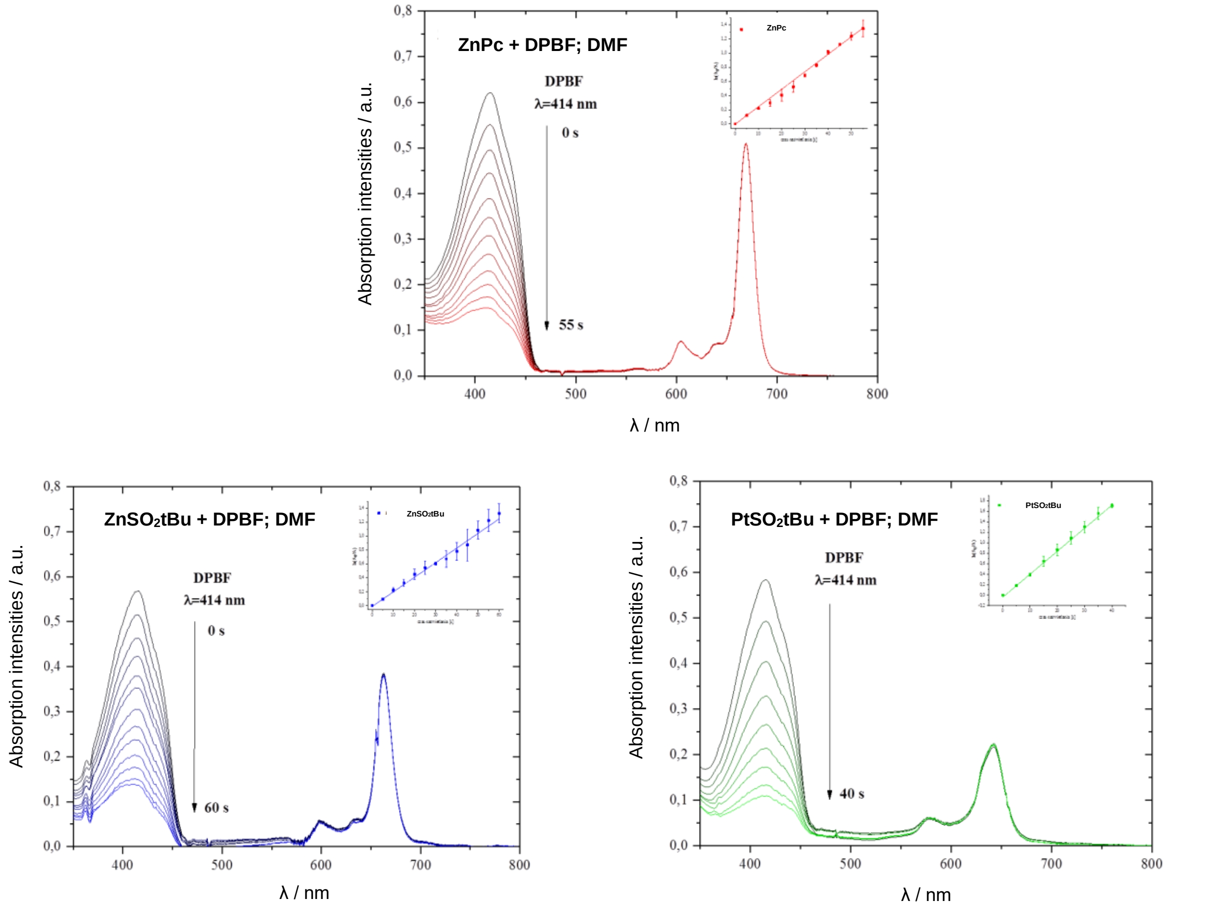

Figure S3 Changes in absorption intensity of DPBF and phthalocyanine mixture in DMF during irradiation, [DPBF] = 6 μM, [**ZnPc**] = [**ZnSO2tBu**] = [**PtSO2tBu**] = 5 μM, cut off filter < 550 nm, light intensity 67 mW/cm^2

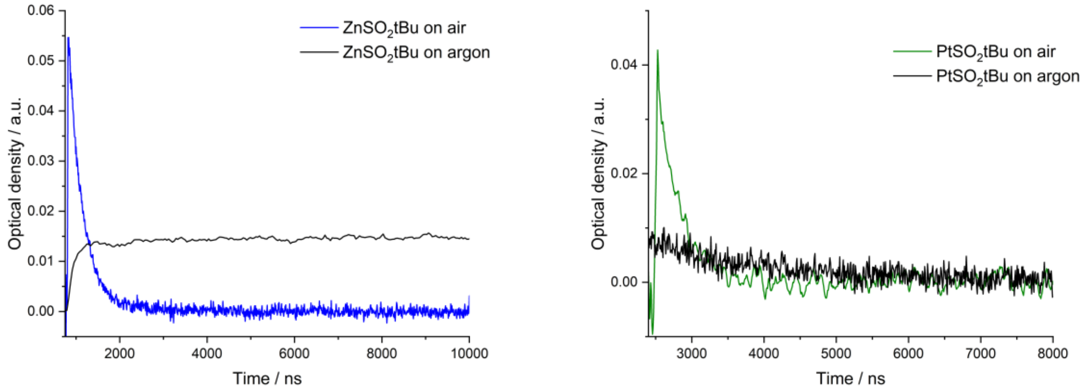

Figure S4 Influence of oxygen on the decay of **ZnSO2tBu** and **PtSO2tBu** triplet state (solutions were purged with argon).

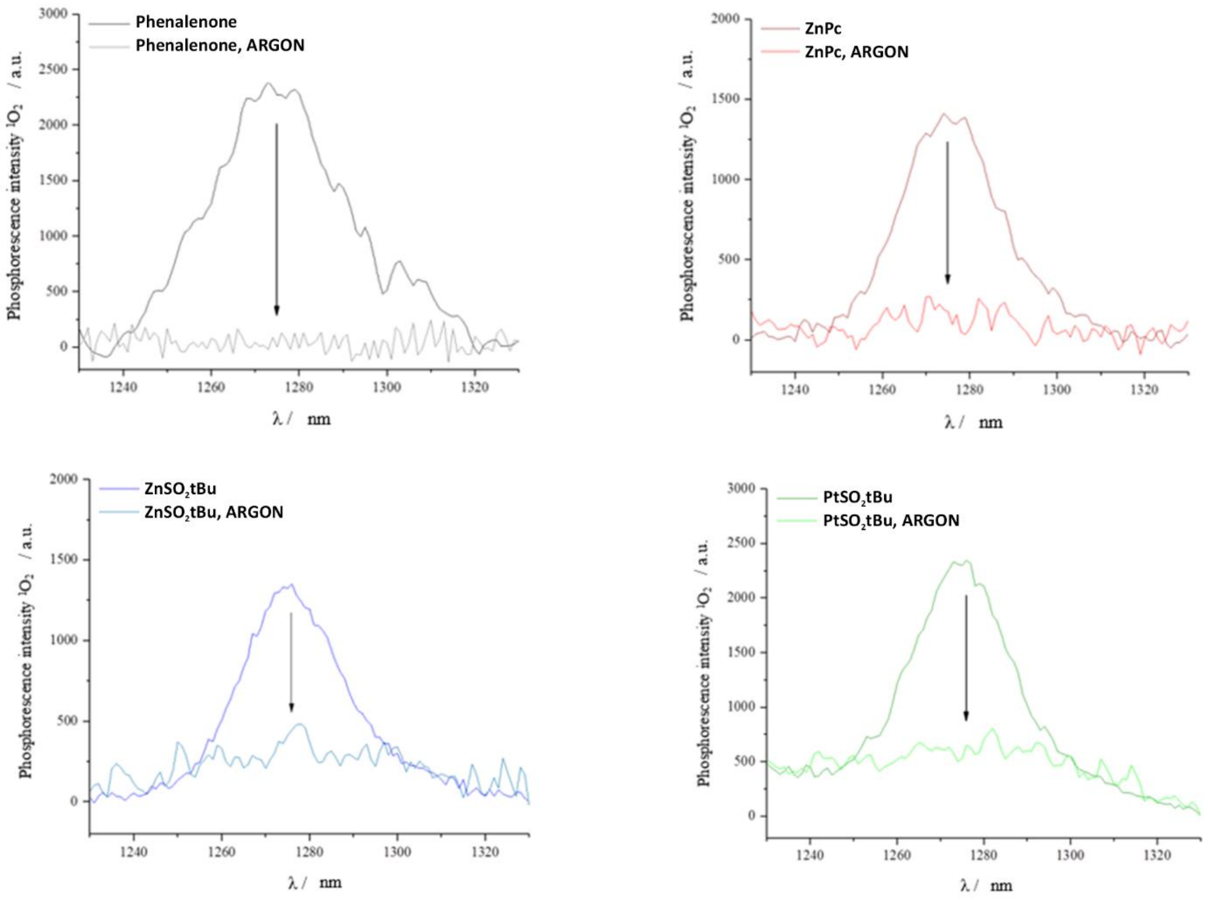

Figure S5 Quenching of $^1O_2$ phosphorescence after purging the DMF solutions with argon (15 minutes).

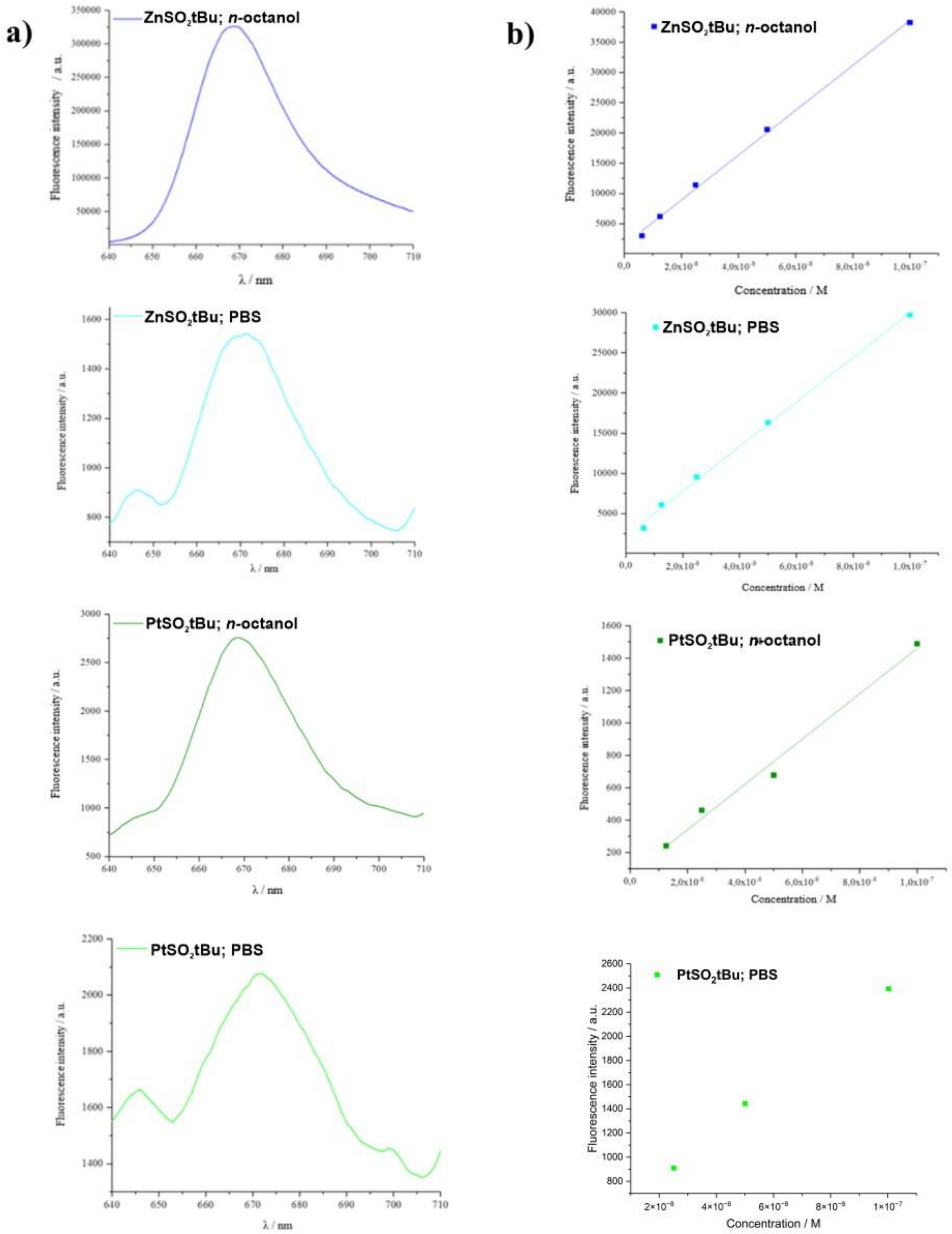

Figure S6 a) Emission spectra of phthalocyanines in different phases (n-octanol, PBS), b) calibration curves in PBS.

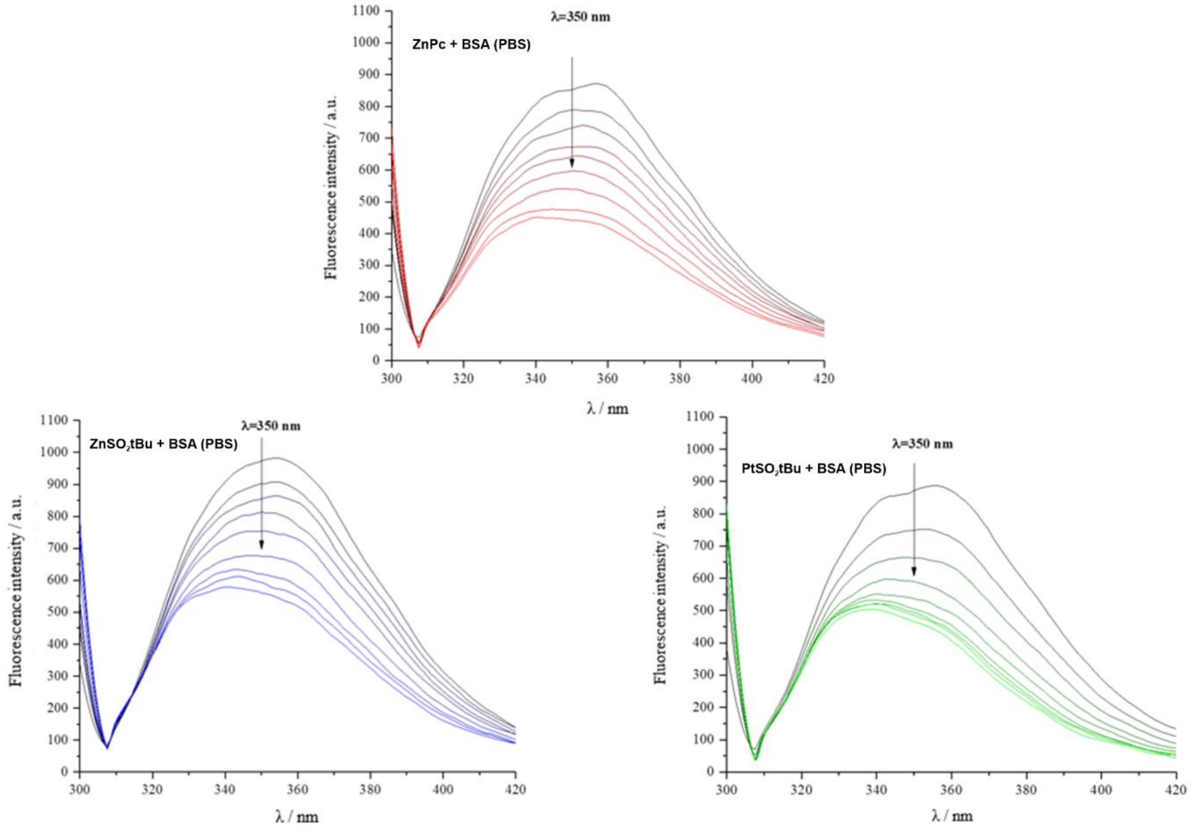

Figure S7 Fluorescence quenching of BSA (1 μM) during titration of **ZnPc**, **$ZnSO_2tBu$** and **$PtSO_2tBu$**.

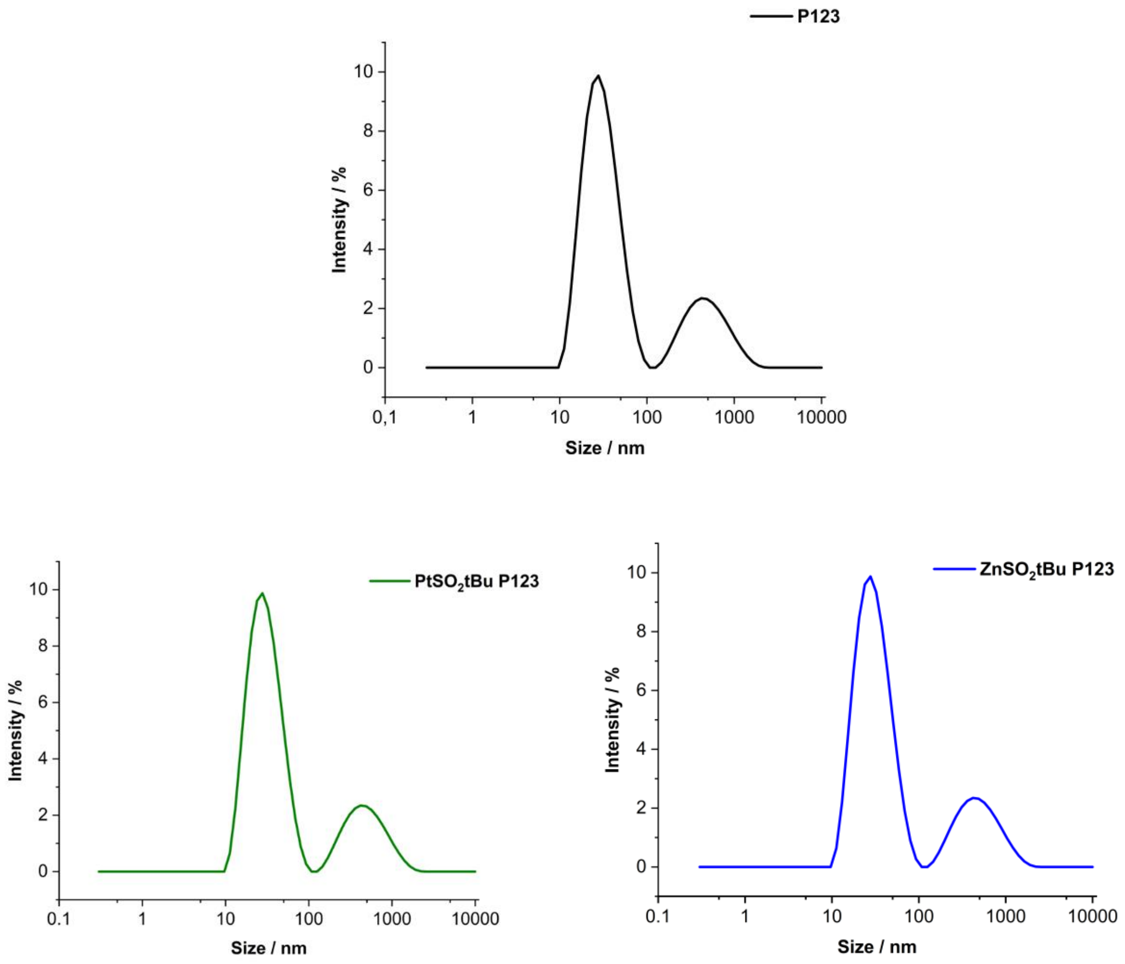

Figure S8 Dynamic Light Scattering for Pluronic® without photosensitizer and with photosensitizers encapsulated in P123.

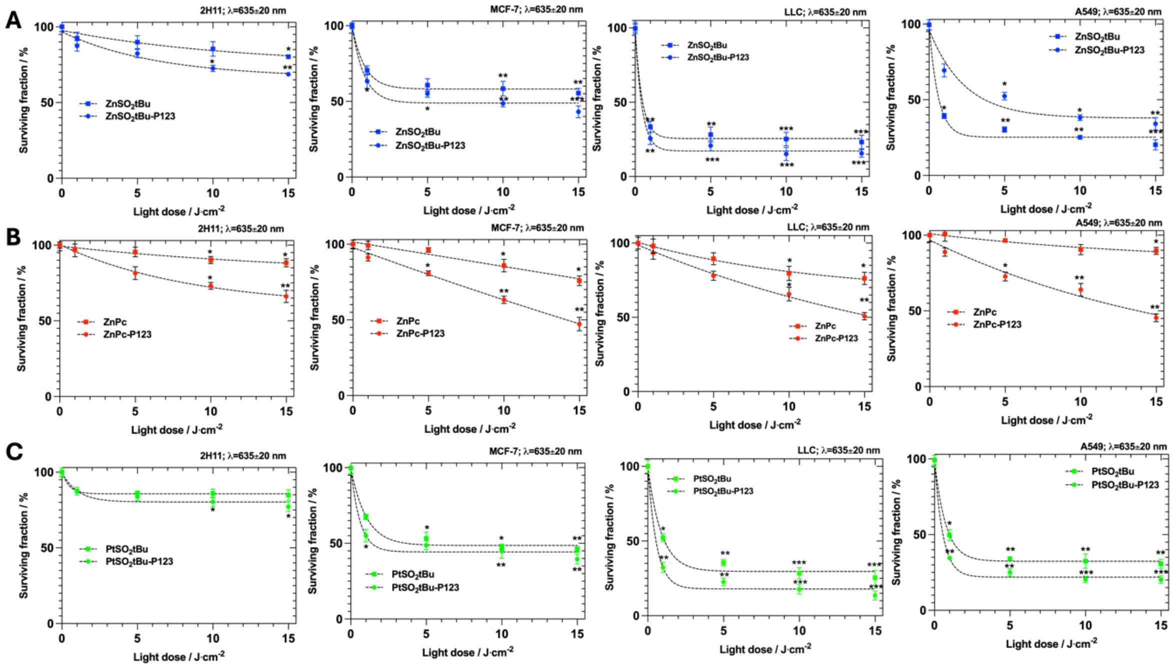

Figure S9 Photodynamic effect of a) $ZnSO_2tBu$, $ZnSO_2tBu$-P123, b) ZnPc, ZnPc-P123, c) $PtSO_2tBu$ and $PtSO_2tBu$-P123 against A549, LLC, MCF-7 and 2H11 cells. The asterisks denote *p*-values < *0.05, **0.01 compared to control.

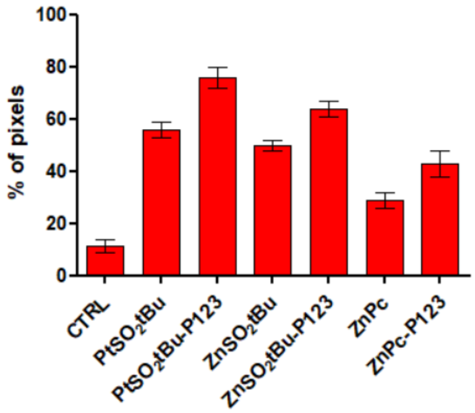

Figure S10 The red pixels quantification based on the images registered for organoids after photodynamic effect with investigated phthalocyanines.

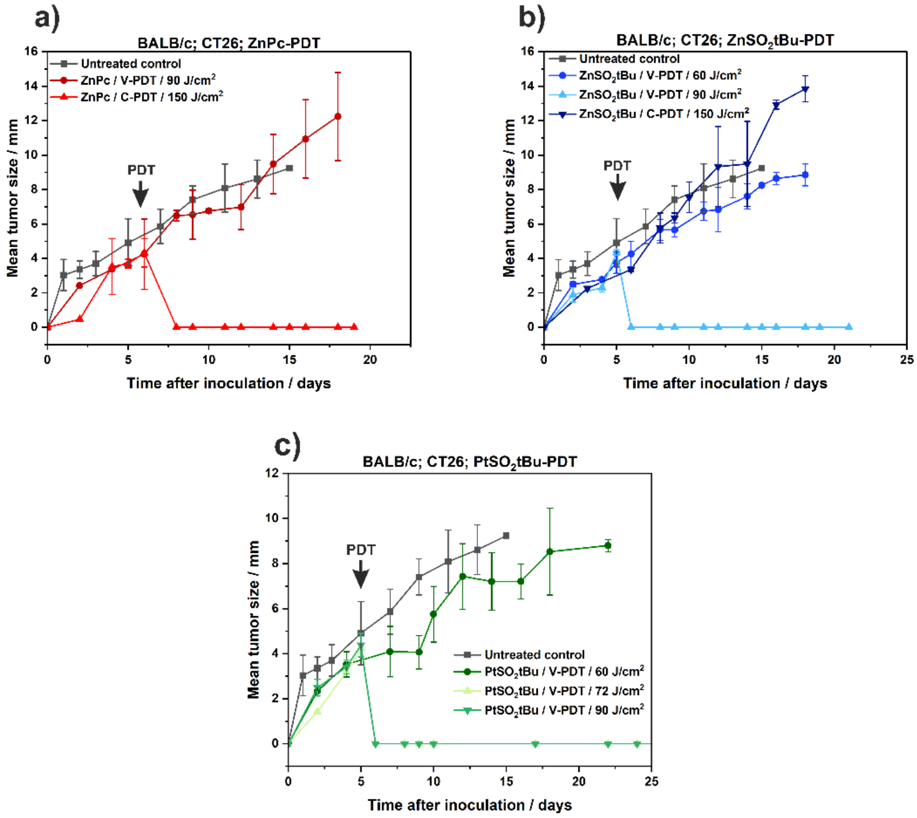

Figure S11 Kinetic curves of CT26 tumors growth for a) ZnPc-P123-PDT, b) $ZnSO_2tBu$-P123-PDT, c) $PtSO_2tBu$-P123, with highlighted moment of PDT treatment. Mean tumor size was measured as cube root of the three-diameter product ((a*b*c)^(1/3)).

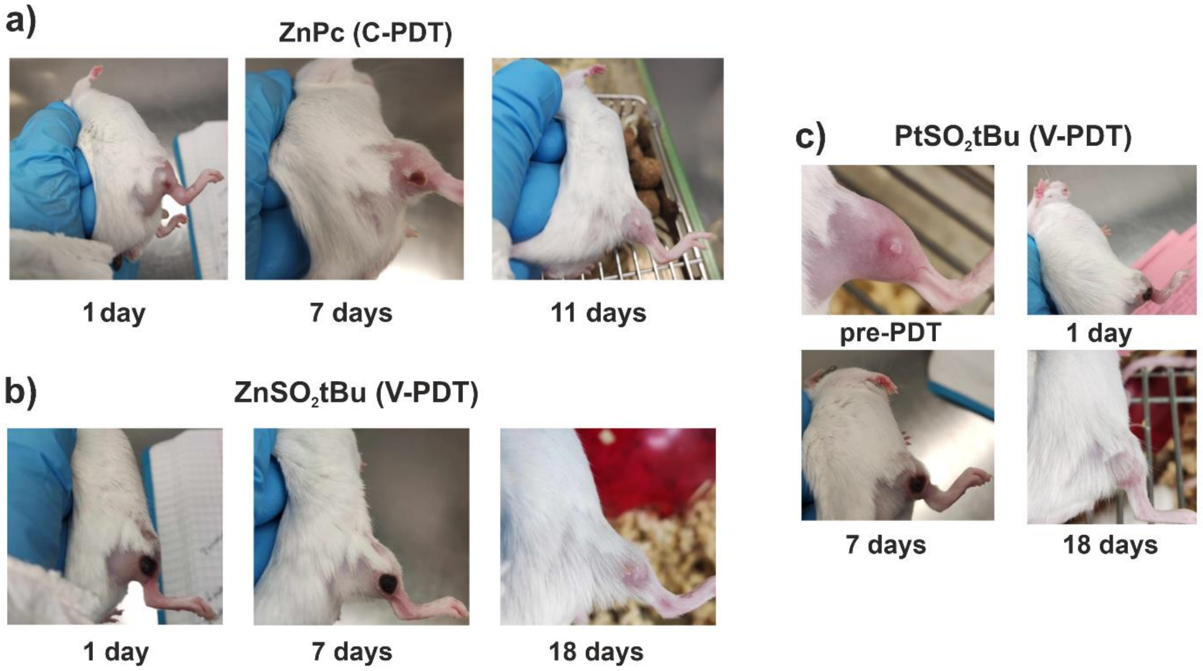

Figure S12 Observations of changes in tumor 1 day after, 7 days after and 18 days after a) ZnPc C-PDT, b) $ZnSO_2tBu$ V-PDT, c) $PtSO_2tBu$ V-PDT, showing the development of local inflammation, formation of a scab and a healthy tissue regeneration.

Table S1 Observations of tumor growth after inoculation of CT26 cells into BALB/c mice, with observation of changes in tumor area post-PDT for **ZnPc** (C-PDT, V-PDT), **$ZnSO_2tBu$** (C-PDT, V-PDT / 60 J/cm^2, V-PDT / 90 J/cm^2), and **$PtSO_2tBu$** (V-PDT / 60 J/cm^2, V-PDT / 72 J/cm^2, V-PDT / 90 J/cm^2)

Time after inoculation/days	ZnPc							$ZnSO_2tBu$												$PtSO_2tBu$								
	C-PDT				V-PDT			C-PDT			V-PDT / 60 J/cm^2			V-PDT / 90 J/cm^2				V-PDT / 60 J/cm^2			V-PDT / 72 J/cm^2			V-PDT / 90 J/cm^2		
	Successful	SD	Unsuccessful	SD	Successful	Unsuccessful	SD	Successful	Unsuccessful	SD	Successful	Unsuccessful	SD	Successful	SD	Unsuccessful	SD	Successful	Unsuccessful	SD	Successful	SD	Unsuccessful	Successful	SD	Unsuccessful
0	0	0	0	0	-	0	0	-	0	0	-	0	0	0	0	0	0	-	0		0	0	-	0	0	-
2	0.467	0	2,084	1,581	-	2,426	0	-	2,261	0	-	2,496	0,140	1,891	0,412	1,891	0	-	2,331	0,037	1,424	0	-	2,490	0,370	-
4	3.527	1.621	3,354	1,379	-	3,374	0	-	3,363	0,114	-	2,777	0,016	2,306	0,264	2,306	0	-	3,526	0,561	3,196	0	-	3,406	0,310	-
5	3.701	0.249	4,047	0,183	-	4,254	2,044	-	5,770	0,873	-	3,758	0,620	4,346	0,160	4,346	0	-	4,09	1,121	PDT		-	PDT		-
6	PS injection		PS injection	0,296	-	6,481	0,309	-	PS injection 6,351	1,784	-	3,758		PDT		PDT		-	4,07	0,744	edema, erythema		-	edema, erythema		-
8	PDT		PDT	1,169	-	PDT 6,542	1,430	-	PDT 7,554	0,892	-	PDT 5,664	0,614	edema, erythema		edema		-	PDT	1,682	necrosis, erythema		-	extensive necrosis		-
9	edema, inflammation		9,071	0,069	-	6,761	1,252	-	9,338	2,324	-	5,658	0,402	necrosis, erythema		necrosis		-	7,43	1,452	scab		-	extensive necrosis		-

11	necrosis		11,706	0,333	-	6,981	1,305	-	9,486	2,471	-	6,740	0,529	scab formation		scab formation		-	7,20	1,276	scab		-	extensive necrosis		-
12	scab formation		13,353	0,719	-	9,484	1,715	-	12,938	0,267	-	6,835	1,290	scab		scab formation		-	7,21	0,779	healthy tissue regeneration		-	healthy tissue regeneration		-
14	scab		euthanasia	0,0	-	10,940	2,276	-	13,856	0,754	-	7,606	0,739	scab		scab formation		-	8,53	1,928	Recovery		-	healthy tissue regeneration		-
15	scab				-	12,246	2,556	-	euthanasia		-	8,255	0,048	scab		tumor recurrence		-	8,80	0,272			-	healthy tissue regeneration		-
16	scab				-	euthanasia	0,000	-			-	8,645	0,362	scab		6,600		-	euthanasia				-	healthy tissue regeneration		-
18	healthy tissue regeneration				-			-			-	8,857	0,636	scab		7,035		-					-	recovery		-
19	recovery				-			-			-	euthanasia		tissue regeneration		6,751		-					-			-
21					-			-			-			recovery		euthanasia		-					-			-

Characterization spectra for ZnSO2tBu

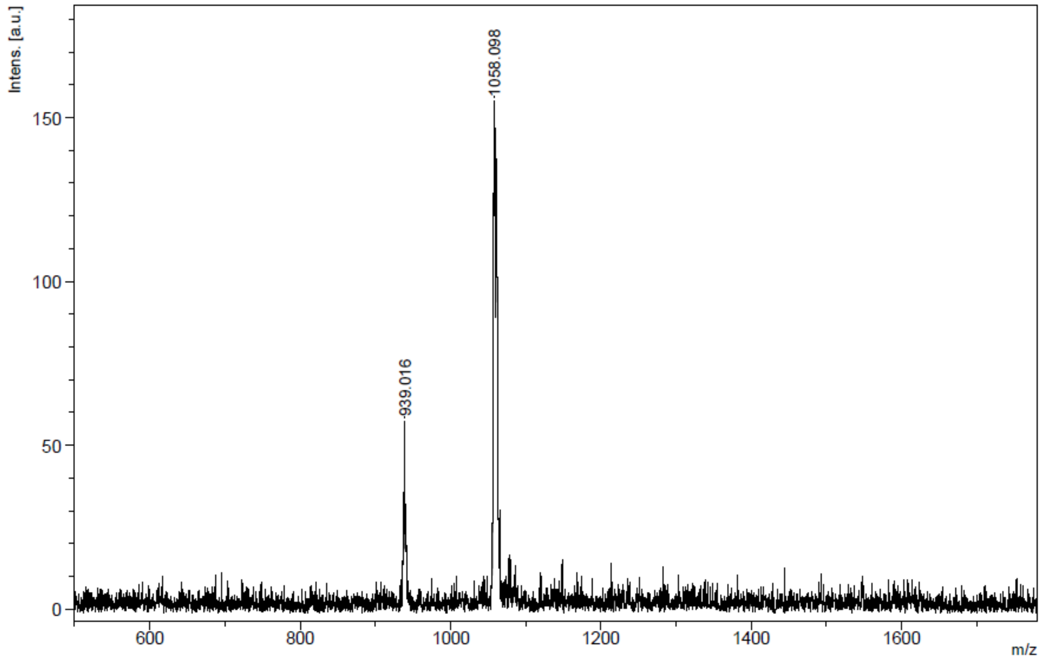

Figure S13. MALDI-MS spectrum of **ZnSO2tBu** (matrix: DIT)

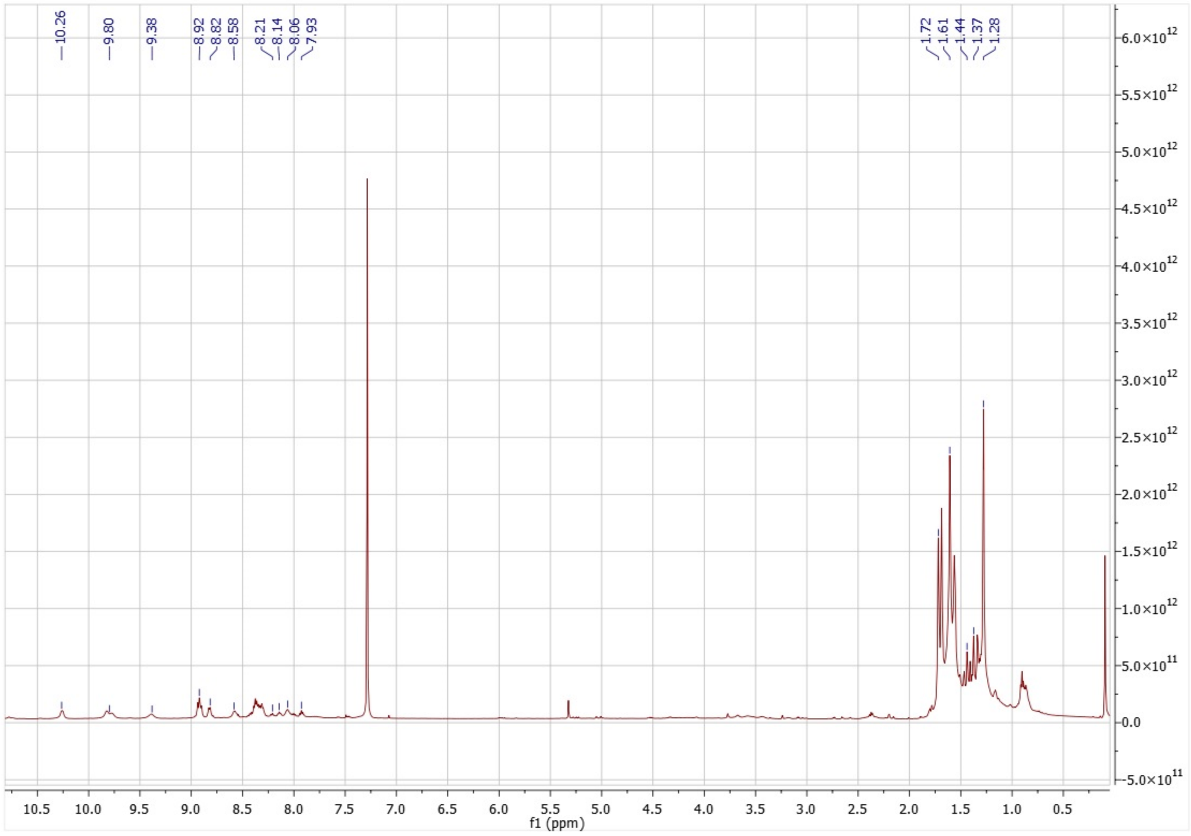

Figure S14. ^{1}H NMR spectrum of **ZnSO2tBu** ($CDCl_3$)

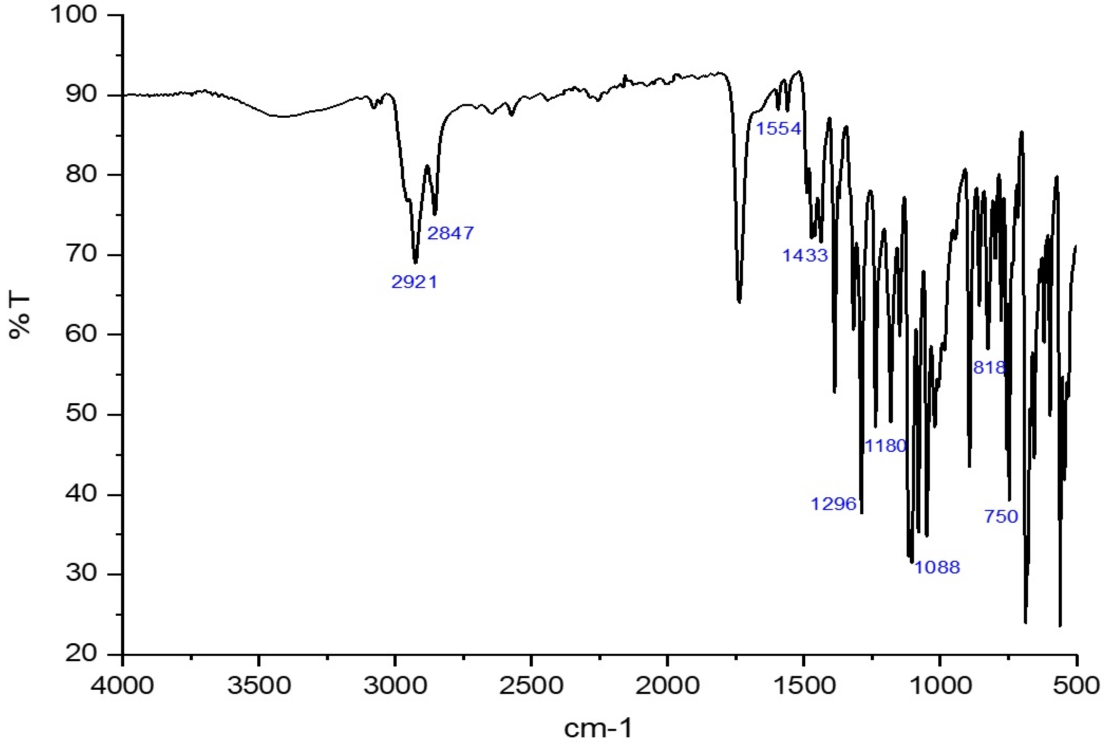

Figure S15. FT-IR spectrum of **$ZnSO_2tBu$**

Characterization spectra for $PtSO_2tBu$

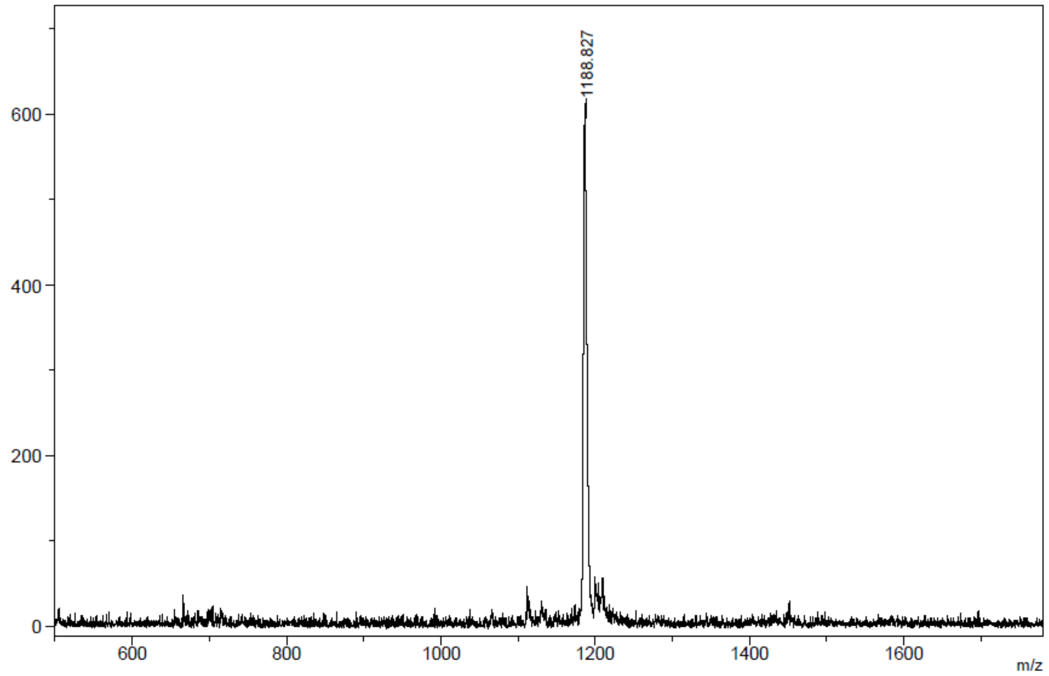

Figure S16. MALDI-MS spectrum of **$PtSO_2tBu$** (matrix: DIT)

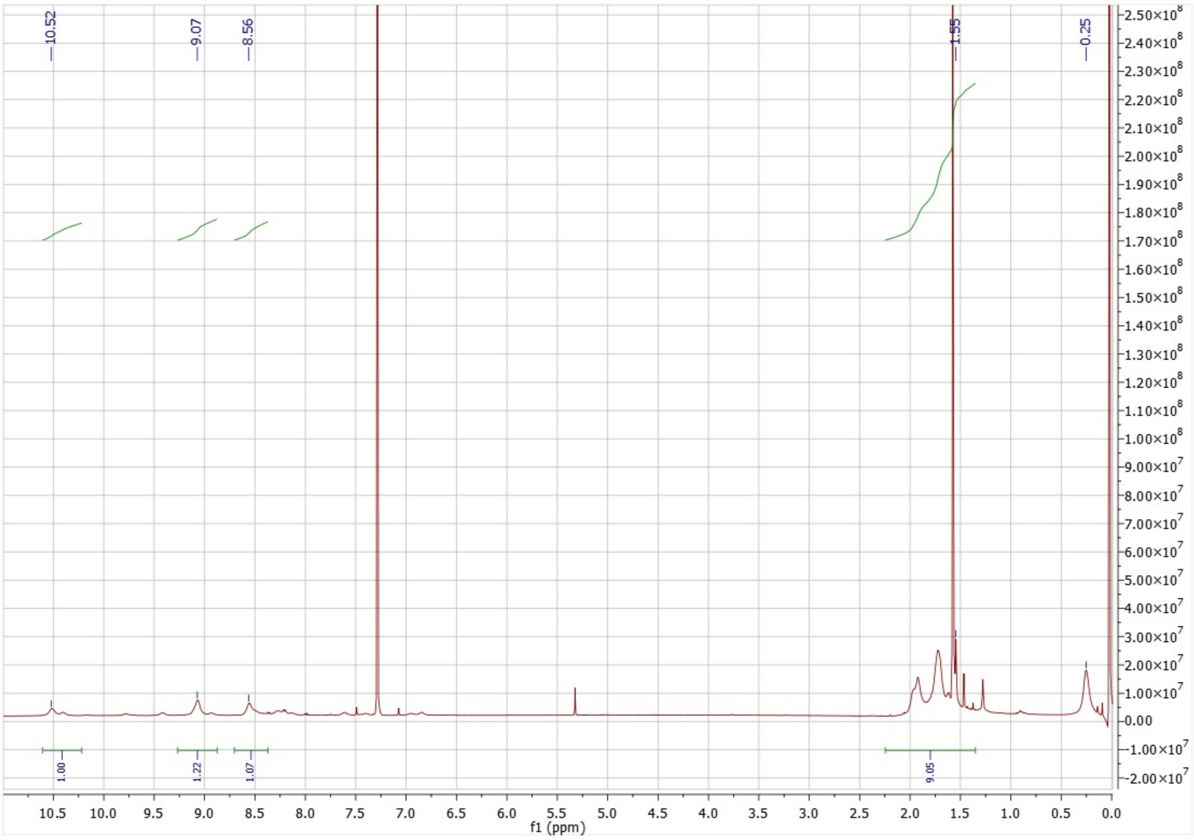

Figure S17. ^{1}H NMR spectrum of **$PtSO_2tBu$** ($CDCl_3$)

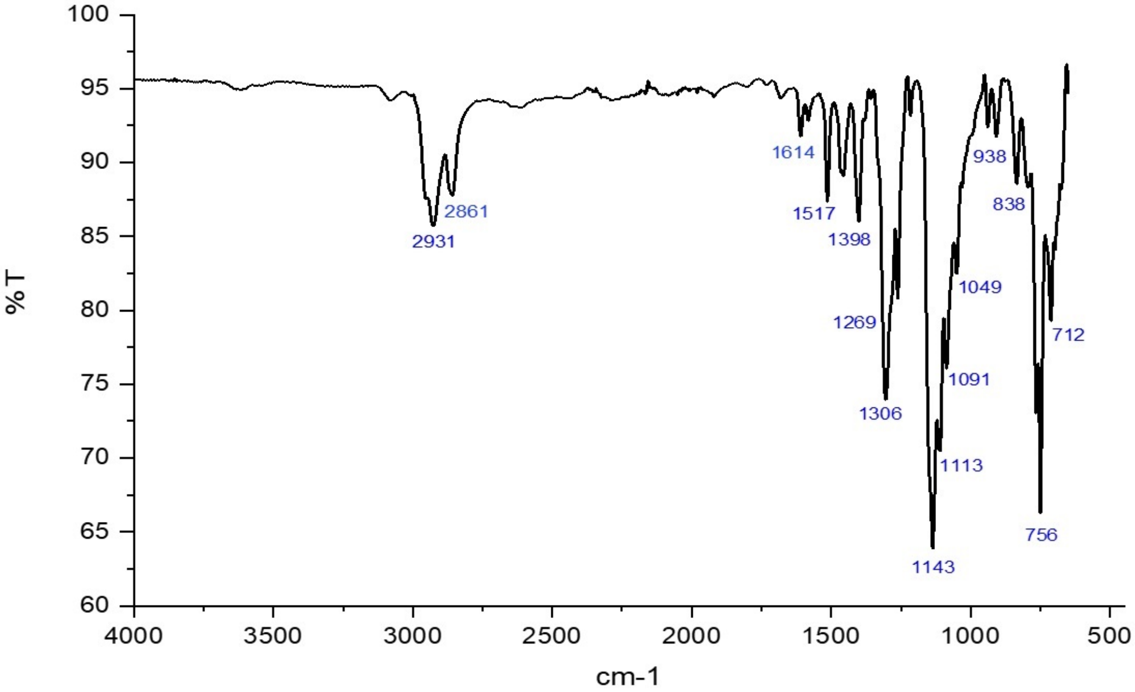

Figure S18. FT-IR spectrum of **$PtSO_2tBu$**
